# Expanding amide bond formation with CaLB-BOP: from sterically hindered substrates to aqueous and micellar media

**DOI:** 10.1039/d6ra00592f

**Published:** 2026-03-18

**Authors:** Katarina Zlatić, Antonija Ožegović, Nikola Maraković, Anamarija Knežević

**Affiliations:** a Division of Organic Chemistry and Biochemistry, Ruđer Bošković Institute Zagreb Croatia Anamarija.Knezevic@irb.hr; b Division of Toxicology, Institute for Medical Research and Occupational Health Zagreb Croatia

## Abstract

Amide bond formation remains a cornerstone transformation in the synthesis of biologically active molecules, yet efficient methods for converting non-activated ethyl esters into amides under mild conditions remain limited. This work investigates the applicability of a one-pot protocol that combines *Candida antarctica* lipase B (CaLB)-mediated ester hydrolysis with phosphonium-based amidation (CaLB-BOP method) for amide synthesis. A set of esters and amines are examined *via* the CaLB-BOP method and benchmarked against classical base-catalyzed and CaLB-catalyzed aminolysis. Molecular docking is employed to probe substrate orientation within the CaLB active site, enabling correlation with experimental ester hydrolyzability and aminolysis reactivity. The CaLB-BOP method demonstrates markedly enhanced efficiency for sterically hindered and less reactive substrate combinations, overcoming limitations of classical enzymatic aminolysis that requires simultaneous binding of ester and amine. The method is also evaluated in aqueous and micellar media, where reactions proceed cleanly, require minimal purification, and display enhanced yields at increased stirring rates and surfactant concentrations. Overall, the CaLB-BOP method provides a practical and mild approach to amide bond formation, especially valuable for sterically demanding substrates and aqueous-phase synthesis.

## Abstract 2

CaLB-BOP method enables efficient amide bond formation from non-activated esters and amines under mild conditions. It outperforms classical enzymatic aminolysis for sterically hindered substrates and operates effectively in water and micellar media.

## Graphical abstract

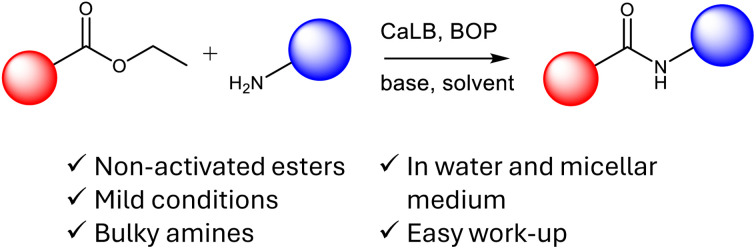

## Introduction

Amide bonds are among the most ubiquitous linkages in organic chemistry, found in numerous natural organic compounds, biologically active synthetic compounds, and polymeric materials. In nature, amide bonds are the fundamental links that connect amino acid residues to form peptides and proteins. Beyond their biological relevance, amide-containing molecules are widely used in the pharmaceutical industry as active substances in drugs.^1^ In fact, the formation of amide bonds represents one of the most frequently employed synthetic transformations in medicinal chemistry.^2–4^ Consequently, amide bonds are prevalent in both small organic molecules and complex macromolecules.^5^

Despite the large number of available methods for amide bond formation, there is still a need to develop new and improved synthetic methodologies.^5–9^ Current efforts focus on increasing reaction efficiency, employing available and cheap catalysts and reagents, reducing reaction times, implementing green solvents, simplifying the processing of the reaction, broadening the substrate scope, *etc.* The most common method for amide bond formation involves the reaction of a carboxylic acid with an amine facilitated by one of many coupling reagents. In order for the carboxylic acid to readily react with the amine, the –OH group is typically activated by conversion into more reactive acyl halides, azides, acylimidazoles, anhydrides or activated esters.^1,7,8^ While activated esters are known to readily undergo aminolysis under mild conditions, simple nonactivated esters (*e.g.*, methyl and ethyl esters) are generally unreactive toward most amines unless subjected to harsh conditions.^10,11^ This property leads to the use of these esters as stable carboxyl function protecting groups in intermediates during multistep organic syntheses.^12^ In such cases, the amide formation step is often performed at a late stage of the synthetic pathway.

We previously reported the preparation of an amide bond in *N*-(3-azido-1-phenylpropyl)-2-hydroxyiminoacetamide from an ethyl ester and an amine, employing a one-pot system that combines an enzyme (*Candida antarctica* lipase B, CaLB) with a condensation reagent (benzotriazolyloxytris(dimethylamino)phosphonium hexafluorophosphate, BOP).^13^ In this study, we apply the concept of decoupling ester hydrolysis from the amidation step to enable reactions with sterically hindered amines, which are challenging for conventional enzymatic aminolysis, as well as reactions in micellar media and water. The method is systematically compared with both base-catalyzed and classical CaLB-catalyzed aminolysis to evaluate its performance and limitations. Experimental results are complemented by molecular docking studies, which probe the binding orientation of substrates within the enzyme's active site and help rationalize reactivity trends. Finally, we demonstrate the applicability of CaLB-BOP method under environmentally friendly conditions, specifically in aqueous and micellar media, showing that the decoupled hydrolysis–amidation approach remains effective in these alternative reaction media.

## Results and discussion

### CaLB-BOP in organic solvents

**Fig. 1:**
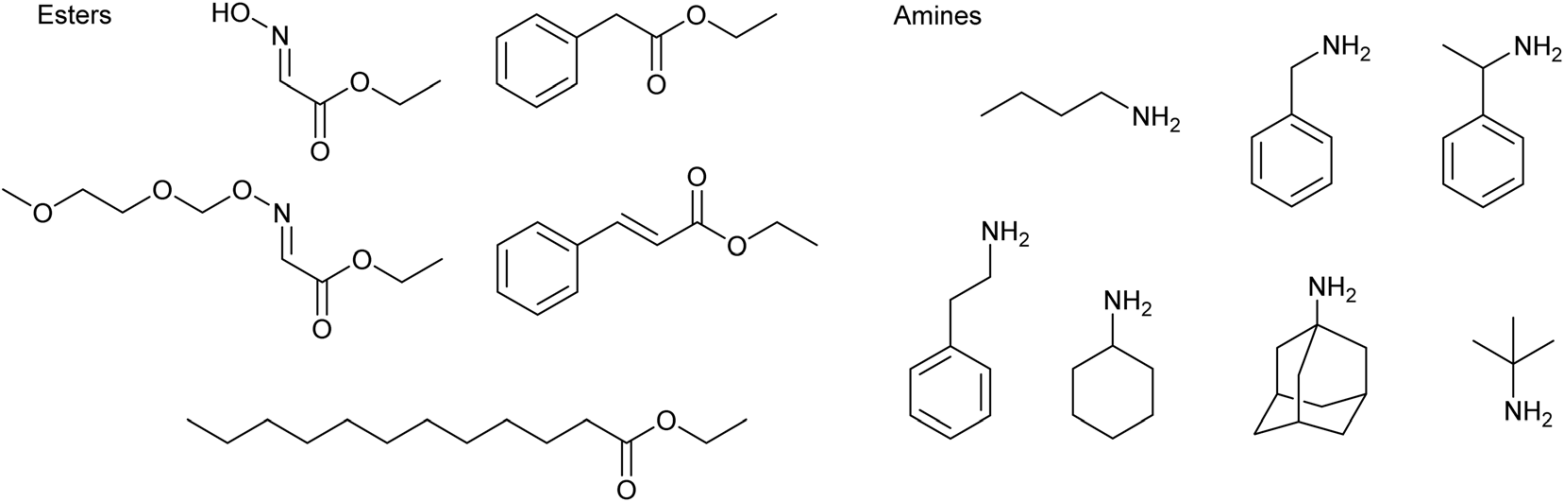
Structures of selected esters and amines.

The CaLB-BOP method was compared with both the conventional base-catalyzed aminolysis and classical enzyme-catalyzed reaction (CaLB under anhydrous conditions in a dry organic solvent), with all reactions conducted in organic media. To investigate the substrate scope of the method, a range of esters and amines was selected (Fig. 1). The ester set included ethyl glyoxylate oxime and its MEM protected analog, previously used in our earlier work,^13^ as well as bulkier esters such as ethyl phenylacetate, ethyl cinnamate and ethyl dodecanoate. In addition to steric effects, these esters also differ in reactivity—oxime-bearing esters are substantially more reactive toward aminolysis than ethyl phenylacetate, ethyl cinnamate and ethyl dodecanoate, primarily due to the electron-withdrawing effect of the oxime group, which increases the electrophilicity of the carbonyl carbon. The amine set ranged from relatively simple substrates, such as *n*-butylamine, benzylamine, and 2-phenylethylamine, to more sterically demanding ones, including 1-phenylethylamine, 1-adamantylamine, cyclohexylamine, and *tert*-butylamine.

First, amide bond formation was attempted *via* a standard base-catalyzed method using the selected esters and amines (Table 1, top). In our previous paper, we tested different conditions for this reaction, and here we applied the conditions that had provided the best results – the reactions were heated at 70 °C for 48 hours in ethanol with triethylamine as the base.^13^ As expected, less reactive esters ethyl phenylacetate, ethyl cinnamate and ethyl dodecanoate did not yield any products with the tested amines. Slightly more reactive esters, ethyl glyoxylate oxime and its MEM protected analog, reacted only with sterically unhindered amines in a moderate way. However, the high temperature and prolonged reaction times resulted in complex mixtures with numerous byproducts, complicating purification and reducing the practicality of this method.

**Table 1:** The comparison of methods for amide bond formation between selected esters and amines. Yields (%) are presented in the table

	Ethyl glyoxylate oxime	Ethyl glyoxylate MEM-oxime	Ethyl phenylacetate	Ethyl cinnamate	Ethyl dodecanoate
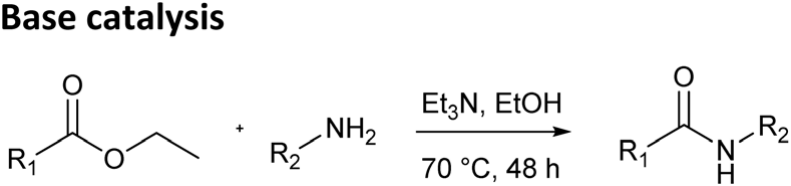					
*n*-Butylamine	51	55	0	0	0
Benzylamine	36	47	0	0	0
1-Phenylethylamine	0	0	0	0	0
2-Phenylethylamine	22	41	0	0	0
1-Adamantylamine	0	0	0	0	0
Cyclohexylamine	11	23	0	0	0
*tert*-Butylamine	0	0	0	0	0

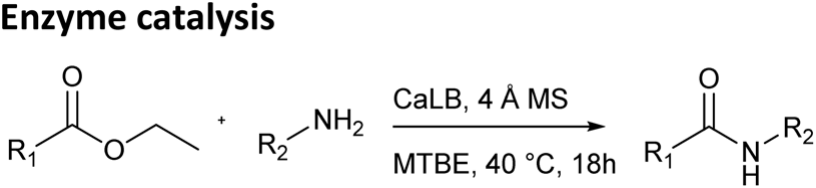					
*n*-Butylamine	99	85	79	46	90
Benzylamine	90	61	65	40	73
1-Phenylethylamine	58	35	21	6	46
2-Phenylethylamine	92	77	99	74	90
1-Adamantylamine	0	0	0	0	0
Cyclohexylamine	29	33	10	6	25
*tert*-Butylamine	0	0	0	0	0

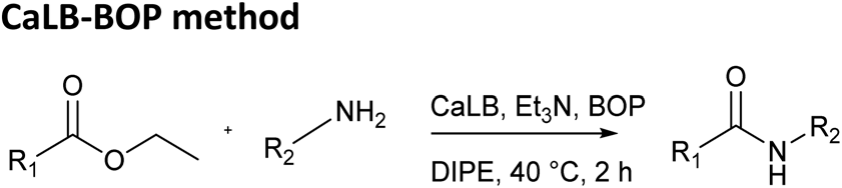					
*n*-Butylamine	11^a^	37	99	77	76
Benzylamine	16^a^	31	98	57	62
1-Phenylethylamine	/b	45	99	57	40
2-Phenylethylamine	/b	64	90	62	70
1-Adamantylamine	0^a^	11	58	31	34
Cyclohexylamine	0^a^	41	50	59	71
*tert*-Butylamine	/b	58	70	61	55

a The reaction was performed at room temperature.

b The reactions with ethyl glyoxylate oxime were not pursued further due to the preliminary results presented in the table.

Next, the same reactions were carried out using a classical enzyme-catalyzed method with CaLB in an organic solvent (Table 1, middle), under reaction conditions that we had previously used for enzymatic catalysis.^13–15^ The active site of the enzyme is spatially restricted, which enables its use for kinetic resolution and the preparation of enantiomerically rich substrates. On the other hand, the limited size of the active site prevents the binding of sterically hindered substrates or limits their access to the necessary functional groups within the enzyme. CaLB is a widely used biocatalyst for the kinetic resolution of primary amines and is also an effective tool for amide bond formation.^16–18^ These reactions are typically performed under strictly anhydrous reaction conditions using molecular sieves, as even trace amounts of water can hydrolyze the acyl donor (ester).^16,19^

In this method, the steric bulk of both the ester and the amine played a crucial role in determining reaction outcomes due to the size limitations of enzyme's active site. Ethyl glyoxylate oxime and its MEM protected analog reacted with smaller amines yielding products in moderate to high yields. However, as the size of the amine increased, product yields decreased significantly, with no conversion observed for sterically hindered such as 1-adamantylamine and *tert*-butylamine. A similar trend was observed when increasing ester size: ethyl phenylacetate reacted with smaller amines in moderate to high yield, while the bulkier ethyl cinnamate gave lower yields. The aliphatic ethyl dodecanoate, a bulky but less rigid ester, performed similarly to ethyl phenylacetate. All three esters reacted poorly, or not at all, with sterically demanding amines such as 1-phenylethylamine, 1-adamantylamine, cyclohexylamine and *tert*-butylamine.

Finally, the CaLB-BOP method was applied to the same set of esters and amides. The procedure reported in our previous paper was optimized using the reaction between ethyl phenylacetate and *n*-butylamine. Initially, the reaction was conducted by adding the ester, amine, base (Et_3_N), and CaLB simultaneously, with the BOP reagent introduced 2 hours later.^13^ However, it was observed that the yield was identical when BOP was added at the beginning of the reaction. Therefore, this simplified method of adding all components at once was used in all subsequent experiments. The amount of CaLB was varied but had minimal impact on the yield, which was expected since the hydrolysis step is typically rapid in this process. Optimization of the base quantity (Table 2) revealed that using two equivalents of the base provided the best results, and this condition was applied in further experiments.

**Table 2:** The optimization of the CalB-BOP method on the reaction between ethyl phenylacetate and *n*-butylamine

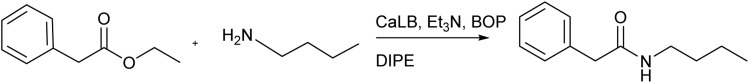			
Et_3_N (equiv.)	Temp.	Time (h)^a^	Yield
1	rt	6	81%
1	40 °C	6	80%
2	40 °C	2	99%
3	40 °C	2	99%

a Reactions were worked up after 6 h or once no ester was detectable in the reaction mixture by TLC.

Once the optimal reaction conditions were established, the CaLB-BOP method was applied in the synthesis of amides from the aforementioned esters and amines (Table 1, bottom). Under these conditions, the distinction between more and less reactive esters became profound. Ethyl glyoxylate oxime afforded only low yields of products with small amines even at room temperature under CaLB-BOP conditions. Attempts to improve yields by separating the hydrolysis and coupling steps were unsuccessful. The low yields are likely due to BOP promoting side reactions involving the unprotected hydroxyl oxime group, which can act as a competing nucleophile. Therefore, this strategy was abandoned for ethyl glyoxylate oxime, and the reactions were performed using its MEM-protected analogue.

In the case of ethyl MEM-protected glyoxylate oxime, higher yields were obtained with bulkier amines, while smaller amines gave somewhat lower yields compared to the classical enzymatic method. Similar trend was observed for the aliphatic ethyl dodecanoate. For the less reactive aromatic esters, ethyl phenylacetate and ethyl cinnamate, the CaLB-BOP method provided the highest yields across almost all tested amines. Notably, it was the only method that successfully yielded amides from sterically demanding amines such as 1-adamantylamine and *tert*-butylamine and gave moderate to high yields for cyclohexylamine and 1-phenylethylamine. This outcome can be attributed to steric constraints—such bulky esters and amines cannot be accommodated within the enzyme's active site in the classical enzymatic reaction. While recent enzymatic amidation methods rely on direct coupling of carboxylic acids with amines—typically by shifting the reaction equilibrium using molecular sieves, ionic liquids, or solvent-free systems under reduced pressure^17,18^—our approach uses the enzyme solely to hydrolyze the ester, while BOP mediates the subsequent amidation. This decoupling allows efficient formation of amides from sterically hindered amines, as the coupling step is no longer constrained by the enzyme's active site.

Finally, it should also be noted that the reaction with the phenanthryl analog of ethyl cinnamate failed under all tested methods. This indicates that the enzyme's active site is not sufficiently spacious to accommodate such a voluminous ester. In addition, reactions of aniline with selected esters produced either no product or only trace amounts of the desired amide, presumably due to the lower nucleophilicity of the amine. These results highlight a limitation of the method, which might be overcome by employing other hydrolytic enzymes with larger active sites and other coupling reagents.

### Docking analysis

**Fig. 2:**
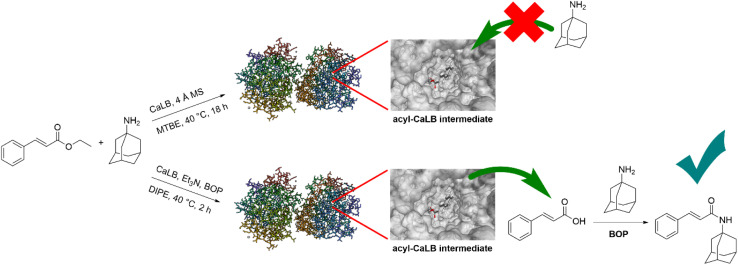
The schematic representation of classical CaLB aminolysis constraint and CaLB-BOP method.

To provide a plausible rationale for the reactivity of different substrates in CaLB-catalyzed aminolysis and the CaLB-BOP method, we conducted a series of molecular docking studies within the binding site of CaLB (Fig. 2). First, the hydrolyzability of esters was analyzed for ethyl phenylacetate, ethyl cinnamate and ethyl (*E*)-3-(phenanthren-9-yl)acrylate. These esters differ in bulkiness, which correlates with the observed experimental differences in the CaLB-BOP method, where hydrolysis is the crucial step. The second part of the docking studies involved analyzing the binding of amines to the acyl–CaLB intermediate of ethyl phenylacetate, providing hypothesis for the underlying structural rationale for the reactivity of different amines in the classical enzyme-catalyzed reaction. The choice of amines was focused on larger amines, such as cyclohexylamine and 1-adamantylamine, which exhibit low or no reactivity, as well as on the notable difference in experimental outcomes between the isomers 2-phenylethylamine and 1-phenylethylamine.

**Fig. 3:**
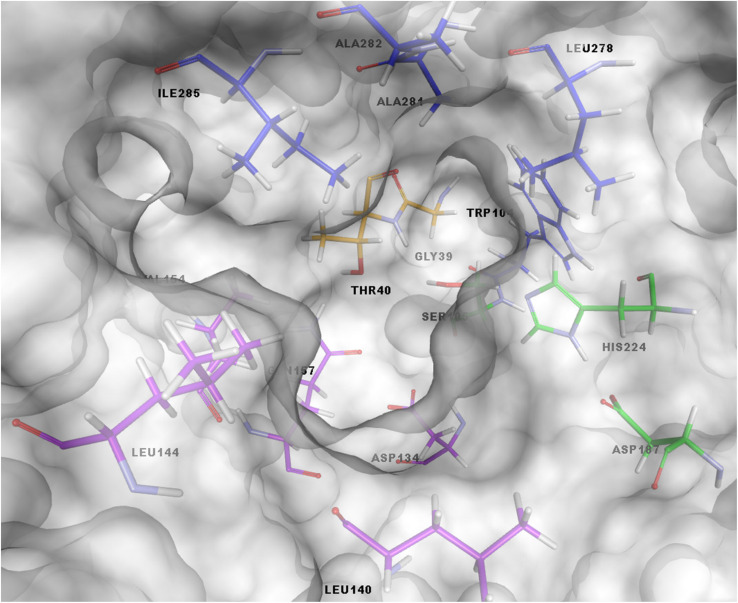
The active site funnel of CaLB (PDB ID: 5A71). The distinct active site residues are shown with stick model where carbon atoms are colored according to their domain origin (acyl pocket/magenta, alcohol pocket/blue, oxyanion hole/yellow, catalytic triade/green).

The binding site of CaLB was analyzed in its open conformation (PDB ID: 5A71),^20^ defined as a sphere (*r* = 12.1 Å) surrounding a 10 Å × 4 Å wide and 12 Å deep narrow channel, as measured from the O_γ_ of Ser105 from the catalytic triad (Ser105-His224-Asp187) to the surface (Fig. 3).^21^ The active site comprises two binding domains: one for the acyl moiety of the ester (more voluminous; defined by Gln106, Asp134, Thr138, Leu140, Leu144, Val154, Gln157, Ile189, and Val190) and one for the alcohol moiety (defined by Thr104, Leu278, Ala281, Ala282, and Ile285), separated by two hydrophobic side chains, Ile189 and Ile285.^21–23^ Additionally, Gly39 and Thr40 form oxyanion hole, stabilizing the negative charge of the tetrahedral intermediate of the ester hydrolysis.^21,24,25^ Thus, when analyzing the hydrolyzability of ester substrate from molecular docking results, the carbonyl oxygen of the ester should be positioned approximately within hydrogen-bonding distance of Gly39 and/or Thr40, and its alcohol oxygen should be within hydrogen-bonding distance from the *N*_ε_ of His224 to enable proton transfer from the catalytic histidine to the alcohol oxygen.^22,26^ After selecting a representative pose of the ester, we modelled the acyl-CaLB intermediate by covalently joining the carbonyl carbon to the Oγ of Ser105, followed by energy minimization. This modelled intermediate was used as a receptor for amine docking. The reacting amine group must both approach the carbonyl carbon within a distance suitable for nucleophilic attack, *i.e.* 5 Å, and have its hydrogen atoms within hydrogen-bonding distance from the *N*_ε_ of His224 to enable proton transfer.^26^

**Fig. 4:**
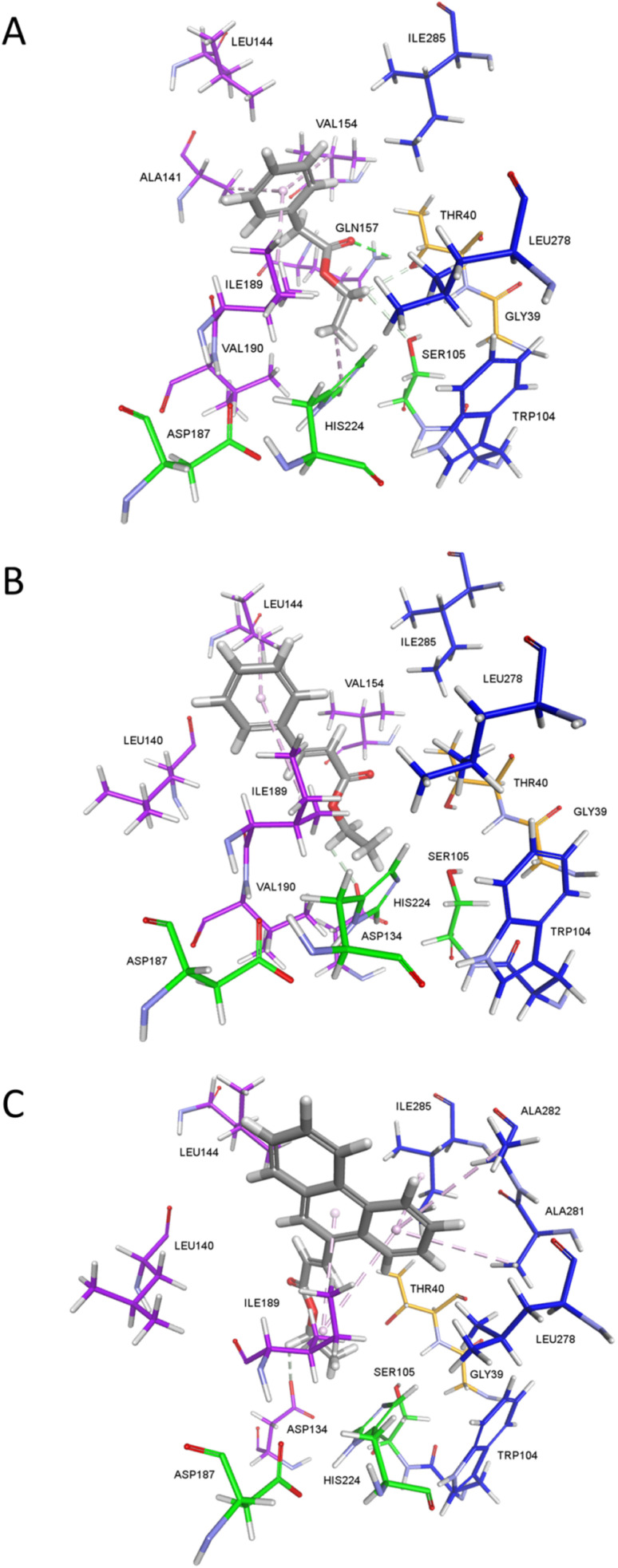
The modeled complexes between CaLB and ethyl phenylacetate (A), ethyl cinnamate (B), and ethyl (*E*)-3-(phenanthren-9-yl)acrylate (C) obtained *via* molecular docking. The non-covalent interactions are shown as dashed lines (pale magenta – aromatic, green – hydrogen bond, conventional, grey – hydrogen bond, other).

Visual inspection of the docking results revealed that, unlike for ethyl phenylacetate and ethyl cinnamate, none of the generated poses of the phenanthryl analog satisfied the requirements for successful hydrolysis. The predicted binding mode of ethyl phenylacetate (Fig. 4A) indicates that some rearrangement of the alcohol part is necessary prior to hydrolysis. While the acyl moiety is predicted to be optimally positioned in the acyl pocked, with its carbonyl oxygen within hydrogen-bonding distance of Thr40, the alcohol part lies outside the alcohol pocket, with the alcohol oxygen over 1 Å beyond the hydrogen-bonding distance from *N*_ε_ of His224, and the ethyl group shielding the carbonyl from nucleophilic attack by Ser105. Ethyl phenylacetate is stabilized through non-bonding interactions, including hydrogen bonds with Gln157, Thr40, and Ser105,^20,21^ and π-alkyl interactions between the benzene ring and acyl pocket residues Ala141, Val154, and Ile189. The predicted binding mode of ethyl cinnamate (Fig. 4B) suggests a similar requirement for rearrangement, albeit it is predicted to be less stabilized only through hydrophobic alkyl or π-alkyl interactions with Val190, Ile189, and Leu144, and a hydrogen bond with Asp134. The latter is notable since Asp134 is known to be engaged in a hydrogen-bond network with Thr40 and Gln157 that dictates the orientation of amphipathic lipid substrates in the CaLB active site.^21^ On the other hand, ethyl (*E*)-3-(phenanthren-9-yl)acrylate (Fig. 4C) is predicted to bind in a conformation which strongly disfavors hydrolysis: the misplaced alcohol part is accompanied with the sterically hindered carbonyl group directed away from the oxyanion hole and into the acyl pocket, while necessary rearrangement is hampered by the bulkiness of the well-stabilized phenanthrene moiety in the acyl part.

**Fig. 5:**
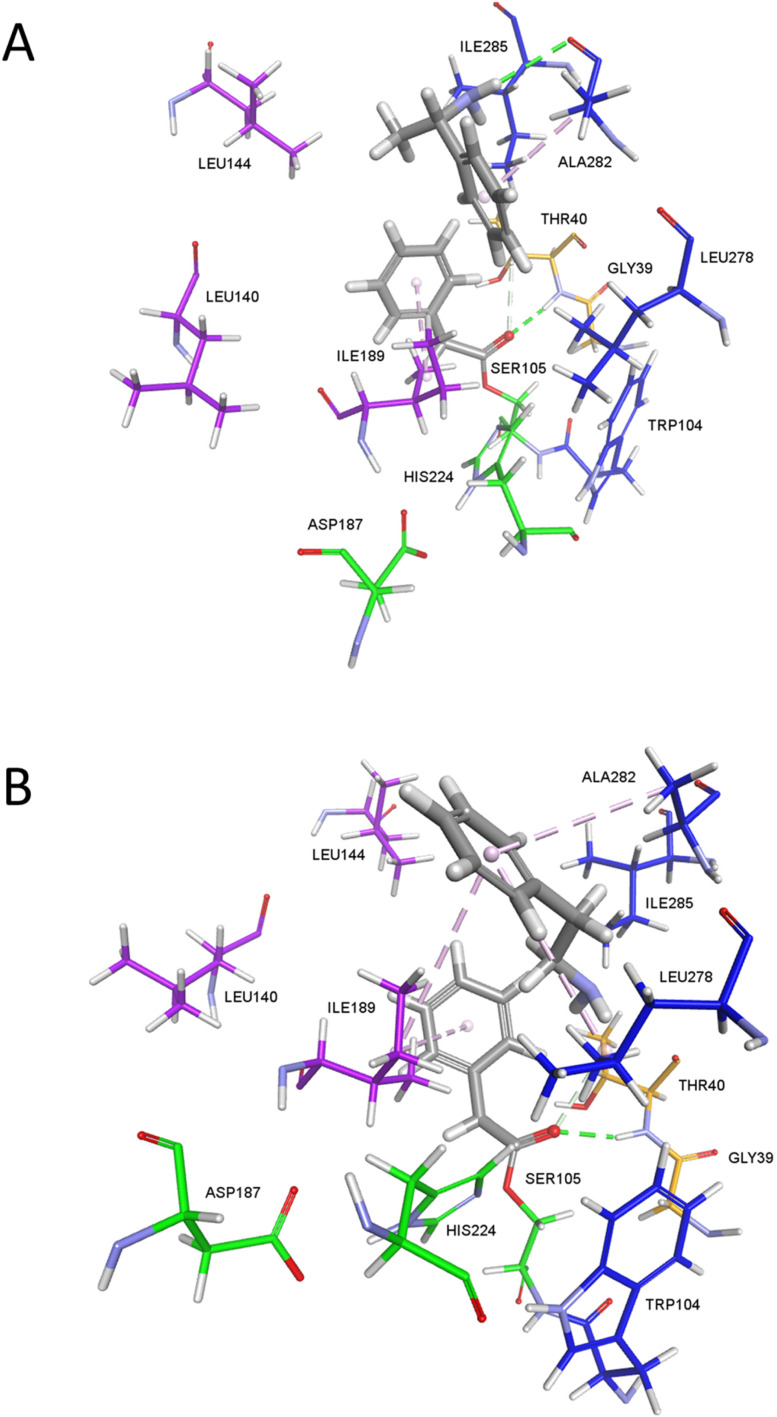
The modeled complexes between CaLB acylated with ethyl phenylacetate and 1-phenylethylamine (A) or 2-phenylethylamine (B) obtained *via* molecular docking. The non-covalent interactions are shown as dashed lines (pale magenta – aromatic, green – hydrogen bond, conventional, grey – hydrogen bond, other).

Docking of amines into the modelled acyl-CaLB intermediate provided hypothesis for the underlying mechanistic rationale for their reactivity in CaLB-catalyzed reactions. For instance, cyclohexylamine and 1-adamantylamine failed to be docked inside the acyl-CaLB intermediate of ethyl phenylacetate, in accordance with their experimentally observed non-reactivity. The relatively unreactive 1-phenylethylamine (Fig. 5A) is predicted to bind in the alcohol pocket, but its amine group is directed away from the carbonyl group and beyond the distance required for nucleophilic attack, while also being stabilized *via* a hydrogen bond and π-alkyl interaction with Ala282. On the other hand, 2-phenylethylamine (Fig. 5B) is also predicted to bind in the alcohol pocked but with its amine group positioned toward the carbonyl at a distance allowing nucleophilic attack. However, its amine protons are outside the hydrogen-bonding distance from *N*_ε_ of His224, suggesting that some conformational rearrangement is necessary to bring the amino group closer for proton transfer and reaction progression. Direct comparison of the predicted binding modes of 1-phenylethylamine and 2-phenylethylamine suggests that the almost T-shape structure of 1-phenylethylamine prevents insertion between the benzene ring of the acyl-CaLB intermediate and the alcohol pocket wall, lined out by residues Leu278, Ala281, and Ala282, which is not the case for the more elongated 2-phenylethylamine.

Overall, the molecular docking studies suggest a clear structural rationale for the experimentally observed reactivity trends for selected substrates in both CaLB-catalyzed ester hydrolysis and aminolysis. The results suggest that ester hydrolysis occurs only when the substrate can adopt a hydrolytically competent orientation within the CaLB active site. In the classical aminolysis pathway, an additional constraint arises from the need for the amine to approach the acyl–enzyme intermediate in a geometry suitable for nucleophilic attack. Together, these insights underscore the central role of steric compatibility and precise positioning within the acyl and alcohol binding pockets in dictating CaLB substrate selectivity and overall reactivity.

### Reactions in micellar media

Finally, we applied the CaLB-BOP method to a model reaction in environmentally friendly solvents – pure water and micellar media. Previously, amide-forming reactions between the corresponding acids and amines in the presence of coupling reagents were shown to proceed in micellar media.^27–29^ These systems offer a greener alternative to traditional organic solvents in organic synthesis.^30–32^ To test the feasibility of our method in such media, we selected the reaction between ethyl phenylacetate and 1-phenylethylamine, which had shown significantly improved yields under CaLB-BOP conditions compared to the classical enzymatic method in organic solvent.

First, it was necessary to identify a suitable base and coupling reagent for the reaction between the corresponding acid and amine in micellar media. The reaction between the phenylacetic acid and 1-phenylethylamine was tested in 2 wt% TPGS in water with Et_3_N, which was used in reactions in organic solvent, and 2,6-lutidine, previously used for amide bond formation in micellar media.^27^ Two coupling reagents were tested for the reaction, namely BOP and EDC. Reactions with Et_3_N failed to produce any product regardless of the coupling reagent, while those with 2,6-lutidine provided products in moderate yields. The reaction with EDC provided product in 38% yield, while with BOP product was obtained in 66% yield. Since reactions in organic solvents were performed with BOP, and this coupling reagent provided higher yield in the test reaction, it was further used for all subsequent reactions in micellar media and water.

Reactions between ethyl phenylacetate and 1-phenylethylamine were tested in both 2% and 6% TPGS-750-M micellar solutions and in pure water (Table 3). When BOP was not added in the solution, only the corresponding acid was isolated, as expected. Likewise, when CaLB was not present in the solution, only the starting ester was detected. This confirms that both the enzyme and the coupling reagent are essential for successful amidation. At room temperature, low yields were obtained in both water and in micellar solutions, with phenylacetic acid still present in the reaction after 4 hours. Yields improved significantly when the temperature was raised to 40 °C. Higher TPGS concentrations and smaller solvent volumes further enhanced the reaction. Both resin-immobilized CaLB (Novozym 435) and lyophilized CaLB performed similarly under these conditions. Immobilized CaLB was used due to more practical reaction processing.

**Table 3:** Testing the reaction between ethyl phenylacetate (1 equiv.) and 1-phenylethylamine (1.1 equiv.) *via* CaLB-BOP method in aqueous and micellar conditions. BOP (1.1 equiv.), 2,6-lutidine (3 equiv.), CaLB (Novozym 435, 150 mg mmol^−1^), 4 h

Entry	Enzyme	Solvent	*V*/mL	Temperature/°C	Yield/%
1	CaLB^a^	2% TPGS	1.5	rt	0^a^
2	—	2% TPGS	1.5	rt	0^b^
3	CaLB	2% TPGS	1.5	rt	29
4	CaLB	H_2_O	1.5	rt	24
5	CaLB	2% TPGS	1.5	40	54
6	CaLB	H_2_O	1.5	40	47
7	CaLB-liof^c^	2% TPGS	1.5	40	47
8	CaLB	2% TPGS	0.75	40	66
9	CaLB	6% TPGS	1.5	40	65
10	CaLB	6% TPGS	0.75	40	76

a Without BOP, only corresponding acid isolated.

b Only starting ester detected.

c Lyophilized enzyme 12 mg.

The first set of experiments was performed on a magnetic stirrer with speeds ranging from 300 to 600 rpm. When the same reactions were carried out on a laboratory shaker (Table 4), obtained yields were much lower. Also, yields improved with the decrease of solvent volume. This indicated that efficient stirring is the crucial parameter for obtaining high yields, as expected for heterogeneous reactions. To explore this further, we performed reaction at two different stirring speeds using the identical magnetic stir bar (Table 4). When the stirring with the magnetic stirrer was at a lower speed, lower yields were obtained, both in water and micellar solution. Higher yields were obtained at the higher speed on the magnetic stirrer. The difference between reactions in water and in micellar solution was consistent across all tested conditions, with micellar solutions improving yields by 10–20%.

**Table 4:** The influence of stirring speed on the yield of the reaction between ethyl phenylacetate (1 equiv.) and 1-phenylethylamine (1.1 equiv.) in aqueous and micellar media. CalB (Novozym 435, 150 mg mmol^−1^), BOP (1.1 equiv.), 2,6-lutidine (3 equiv.), 40 °C, 4 h, solvent volume 1.5 mL, reaction performed in 4 mL vials, for reactions on the magnetic stirrer 8 mm PTFE polygon stir bars with pivot ring were used

Solvent	Rpm	Yield
H_2_O	Shaker – 1000	24
H_2_O	100	49
H_2_O	1000	70
2% TPGS	Shaker – 1000	35
2% TPGS	100	57
2% TPGS	1000	88

Finally, it must be emphasized that reactions performed in both water and micellar media proceed exclusively to the desired amide product, without detectable side-product formation. Furthermore, the reaction work-up is straightforward and involves only simple acid–base extraction. Unreacted acid and amine, as well as byproducts from the coupling reagent, remain in the aqueous layer, while the amide product is isolated exclusively from the organic layer.

## Conclusion

In this study, we demonstrated the efficiency and versatility of the CaLB-BOP method for amide bond formation from non-activated esters and amines under mild conditions. Notably, this approach does not require strictly anhydrous conditions, which are difficult to maintain and typically essential when using CaLB in organic solvents. Compared to the classical enzymatic method, CaLB-BOP showed significantly improved performance for voluminous and sterically hindered substrates. This difference arises because classical CaLB-catalyzed aminolysis requires simultaneous binding of both the ester and the amine in the active site, which we analyzed by docking studies. On the other hand, in the CaLB-BOP method only ester hydrolysis must occur, a step that can be evaluated using the presented docking protocol. While enzymatic catalysis remains more favorable method for the preparation of amides from more reactive esters and smaller amines, the CaLB-BOP method proved especially effective for challenging combinations, such as less reactive esters and bulkier amines, where it was the only approach that consistently yielded the desired amide products. However, both methods are limited to esters that can bind to the CaLB active site in a favorable way, as confirmed by docking studies.

We also evaluated the method's performance in green reaction media – water and micellar solutions. In both cases, the reactions proceeded cleanly, with no side product formation, and required only minimal work-up *via* acid–base extraction. Optimizations revealed that stirring efficiency and micelle concentration significantly influence reaction outcomes, with yields improving at higher stirring rates and TPGS concentrations. Although micellar media generally enhanced yields by 10–20% compared to pure water, both systems proved viable.

Overall, the CaLB-BOP approach offers a mild and practical alternative for amide bond formation. It operates without the need for anhydrous conditions, performs effectively even in aqueous and micellar environments with straightforward reaction workup, and is particularly suitable for sterically hindered amines. These features highlight the applicability of the method for amide synthesis involving sterically demanding amines and reactions performed in aqueous or micellar media.

## Experimental section

### General procedure for CaLB-BOP method

The selected ester (0.68 mmol, 1 equiv.) was dissolved in DIPE (4 mL) with the addition of a few drops of water. The selected amine (0.72 mmol, 1.1 equiv.), triethylamine (200 µL, 1.4 mmol, 2 equiv.), CaLB (100 mg) and BOP (300 mg, 0.68 mmol, 1 equiv.) were then added to the solution. The reaction mixture was stirred at 40 °C for 2 h. The enzyme was filtered off and washed with DIPE and CH_2_Cl_2_. The filtrate was extracted with saturated aqueous NH_4_Cl solution, dried over Na_2_SO_4_, filtered, and evaporated under reduced pressure. The crude residue was purified by silica gel column chromatography (DCM–MeOH, 100 : 1 to 20 : 1, depending on the product) to give the amide product.

### General procedure for CaLB-BOP method in micellar media

Ethyl phenylacetate (57 mg, 0.35 mmol, 1 equiv.) was added to a solution of 1-phenylethyl amine (45 µL, 0.35 mmol, 1 equiv.) in 2% TPGS micellar solution (1.5 mL), followed by 2,6-lutidine (120 µL, 1.0 mmol, 3 equiv.), CaLB (50 mg), and BOP (155 mg, 0.35 mmol, 1 equiv.). The reaction mixture was stirred at 40 °C for 4 h under air. The mixture was extracted with MTBE (3x). The combined organic layers were washed with 1 M HCl (2x) and 2 M Na_2_CO_3_ (2x), dried over Na_2_SO_4_, filtered, and evaporated under reduced pressure. The obtained white solid was the pure amide product.

## Author contributions

K. Z.: investigation, methodology and formal analysis (synthesis in organic solvents, optimization of reaction conditions and characterization of compounds). A. O.: investigation and formal analysis (synthesis in micellar media and water, and characterization of compounds). N. M.: investigation, software and formal analysis (developing and performing docking analysis), visualization. A. K.: investigation and formal analysis (synthesis in organic solvents and micellar solution, characterization of compounds), conceptualization, funding acquisition, supervision, writing – original draft. All authors contributed to the reviewing and editing of the manuscript.

## Conflicts of interest

The authors have no conflicts of interest to declare.

## Supplementary Material

RA-016-D6RA00592F-s001

## Data Availability

The data supporting this article have been included as part of the supplementary information (SI). Supplementary information: synthetic and docking methods, NMR spectra and HRMS spectra. See DOI: https://doi.org/10.1039/d6ra00592f.
