# Supplementary material for: Expanding amide bond formation with CaLB-BOP: from sterically hindered substrates to aqueous and micellar media
Source: RSC Adv. 2026 Mar 18;16(17):15290–9. doi: 10.1039/d6ra00592f (PMC12998939; doi:10.1039/d6ra00592f)
Supplement: RA-016-D6RA00592F-s001 [file RA-016-D6RA00592F-s001.pdf]

Supplementary Information (SI) for RSC Advances.
This journal is © The Royal Society of Chemistry 2026

SUPPLEMENTARY INFORMATION

Expanding Amide Bond Formation with CaLB-BOP: From Sterically Hindered Substrates to Green Reaction Media

*Katarina Zlatić[a], Antonija Ožegović[a], Nikola Maraković[b], Anamarija Knežević[a]**

[a] Division of Organic Chemistry and Biochemistry, Ruđer Bošković Institute, Zagreb, Croatia

[b] Division of Toxicology, Institute for Medical Research and Occupational Health, Zagreb, Croatia

*Corresponding author:

E-mail: Anamarija.Knezevic@irb.hr

Table of Contents

General Methods: 1

Synthesis 2

Docking methods: 5

NMR spectra: 6

HRMS analysis 15

References 24

General Methods:

All reactions in organic solvents were conducted under an argon atmosphere unless stated otherwise. MTBE was dried over activated 4 Å molecular sieves for 48 h. Commercial grade reagents and solvents were used without further purification. Lipase acrylic resin from *Candida antarctica* (Novozym 435, 10000 U/g, recombinant, expressed in *Aspergillus niger*) and TPGS-750-M 2 wt. % in H_2O were obtained from Sigma Aldrich.

TLC was performed on aluminum-baked silica plates (60 F254, Merck) and visualized using either UV light (254 nm) or phosphomolybdic acid reagent. Column chromatography was performed on silica gel (Silicagel 60, 70–230 mesh, Merck) or flash silica gel (Silicagel 60, 230-400 mesh, Merck). ^{1}H and ^{13}C{1H} NMR spectra were recorded on a Bruker AV 300 and 600 spectrometers in $CDCl_3$ or d_6-DMSO. Chemical shifts (δ) are given in ppm and are referenced to TMS or the internal protic solvent. Coupling constants are given in Hz. Chemical purity and reaction progress were monitored by HPLC on a Shimadzu 10A VP HPLC system with a DAD detector. The stationary phase was a Nucleosil 100-5 C18, 250 mm x 4.6 mm column. The following solvent systems were used as mobile phases: H_2O, MeOH, H_3PO_4 (85%) = 90:10:0.5 (A); MeOH (B); Gradient method: 30/100/100/30 %B in 0/20/25/27 min, 1 mL/min. High-resolution mass spectrometry (HRMS) was performed on Agilent 6546 LC/Q-TOF coupled with Agilent 1290 Infinity II HPLC instrument (+ESI).

The products of ethyl phenylacetate and ethyl cinnamate with all the tested amines, specifically *N*-butyl-2-phenylacetamide, *N*-benzyl-2-phenylacetamide, N-(1-phenylethyl)-2-phenylacetamide, *N*-phenethyl-2-phenylacetamide, *N*-((3*s*,5*s*,7*s*)-adamantan-1-yl)-2-phenylacetamide, *N*-cyclohexyl-2-phenylacetamide, *N*-(*tert*-butyl)-2-phenylacetamide, *N*-butylcinnamamide, *N*-benzylcinnamamide, *N*-(1-phenylethyl)cinnamamide, *N*-phenethylcinnamamide, *N*-((3*s*,5*s*,7*s*)-adamantan-1-yl)cinnamamide, *N*-cyclohexylcinnamamide and *N*-(*tert*-butyl)cinnamamide are commercially available compounds. Thus, the NMR spectra of obtained products were compared to online available spectra. (*E*)-*N*-butyl-2-(hydroxyimino)acetamide,[1] (*E*)- *N*-phenethyl-2-(hydroxyimino)acetamide,[1] (*E*)- *N*-cyclohexyl-2-(hydroxyimino)acetamide,[1] (*E*)-*N*-(*tert*-butyl)-2-(hydroxyimino)acetamide,[1] *N*-butyldodecanamide,[2] *N*-(phenylmethyl)dodecanamide,[2,3] *N*-(1-phenylethyl)dodecanamide,[4] *N*-(2-phenylethyl)dodecanamide,[3] *N*-(adamantan-1-yl)dodecanamide,[5] *N*-cyclohexyldodecanamide,[6] *N*-(*tert*-butyl)dodecanamide[7] are previously reported compounds and their spectra were compared to literature spectra.

Synthesis

General procedure for base-catalyzed reaction:

The selected ester (0.68 mmol, 1 equiv.) and amine (0.72 mmol, 1.1 equiv.) were dissolved in EtOH (4 mL), and triethylamine (0.2 mL, 1.4 mmol, 2 equiv.) was added to the solution. The reaction mixture was stirred at 70 °C for 48 h. Ethanol was removed by distillation, and the crude residue was purified by silica gel column chromatography (DCM-MeOH, 100:1 to 20:1, depending on the product) to give the amide product.

General procedure for enzyme-catalyzed reaction:

The selected ester (0.68 mmol, 1 equiv.) was dissolved in dry MTBE (4 mL), and 4 Å molecular sieves (200 mg) and the amine (0.72 mmol, 1.1 equiv.) were added to the solution. The reaction mixture was stirred at 35 °C for 15 min, after which immobilized lipase B from *C. antarctica* (100 mg) was added. The mixture was stirred at 40 °C overnight. The enzyme was filtered off and washed with DIPE and CH_2Cl_2. The filtrate was extracted with saturated aqueous NH_4Cl solution, dried over Na_2SO_4, filtered, and evaporated under reduced pressure. The crude residue was purified by silica gel column chromatography (DCM-MeOH, 100:1 to 20:1 depending on the product) to give the amide product.

(*E*)-*N*-benzyl-2-(hydroxyimino)acetamide

^{1}H NMR (300 MHz, $CDCl_3$) δ 8.66 (s, 1H), 7.54 (s, 1H), 7.39 – 7.21 (m, 5H), 6.83 (br. s, 1H), 4.51 (d, J = 6.0 Hz, 2H). ^{13}C{^{1}H} NMR (75 MHz, $CDCl_3$) δ 162.24, 144.39, 137.38, 128.80, 127.79, 127.75, 43.29. HRMS (ESI) *m/z*, ([M+H]$^+$): calcd. for $C_9H_{11}N_2O_2$: 179.0815, found: 179.0814.

(*E*)-2-(hydroxyimino)-*N*-(1-phenylethyl)acetamide

^{1}H NMR (300 MHz, $CDCl_3$) δ 8.98 (s, 1H), 7.50 (s, 1H), 7.38 – 7.20 (m, 5H), 6.92 – 6.67 (m, 1H), 5.24 – 5.11 (m, 1H), 1.53 (d, J = 6.9 Hz, 3H). ^{13}C{^{1}H} NMR (75 MHz, $CDCl_3$) δ 161.73, 144.22, 142.38, 128.72, 127.56, 126.10, 48.71, 21.68. HRMS (ESI) *m/z*, ([M+H]$^+$): calcd. for $C_{10}H_{13}N_2O_2$: 193.0972, found: 193.0970.

(*E*)-*N*-butyl-2,5,7-trioxa-8-azadec-8-en-10-amide

^{1}H NMR (300 MHz, $CDCl_3$) δ 7.49 (s, 1H), 6.58 (br. s, 1H,), 5.23 (s, 2H), 3.80 – 3.75 (m, 2H), 3.58 – 3.53 (m, 2H), 3.38 (s, 3H), 3.36 – 3.27 (m, 2H), 1.58 - 1.48 (m, 2H), 1.42 – 1.31 (m, 2H), 0.93 (t, J = 7.3 Hz, 3H). ^{13}C{^{1}H} NMR (75 MHz, $CDCl_3$) δ 161.22, 145.16, 98.38, 71.50, 68.17, 58.98, 38.94, 31.43, 19.99, 13.64. HRMS (ESI) *m/z*, ([M+H]$^+$): calcd. for $C_{10}H_{21}N_2O_4$: 233.1496, found: 233.1496.

(*E*)-*N*-benzyl-2,5,7-trioxa-8-azadec-8-en-10-amide

^{1}H NMR (300 MHz, $CDCl_3$) δ 7.55 (s, 1H), 7.40 – 7.21 (m, 5H), 6.90 (br. s, 1H), 5.22 (s, 2H), 4.51 (d, 2H, J = 6.0 Hz), 3.79 – 3.73 (m, 2H), 3.58 – 3.51 (m,2H), 3.36 (s, 3H). ^{13}C{^{1}H} NMR (75 MHz, $CDCl_3$) δ 161.25, 144.95, 137.53, 128.77, 127.92, 127.71, 98.54, 71.54, 68.30, 59.04, 43.25. HRMS (ESI) *m/z*, ([M+H]$^+$): calcd. for $C_{13}H_{19}N_2O_4$: 267.1339, found: 267.1339.

(*E*)-*N*-(1-phenylethyl)-2,5,7-trioxa-8-azadec-8-en-10-amide

^{1}H NMR (600 MHz, $CDCl_3$) δ 7.50 (s, 1H), 7.37 – 7.32 (m, 4H), 7.30 – 7.27 (m, 1H), 6.80 (br. s, 1H), 5.24 (q, J = 7.5 Hz, 2H), 5.21 – 5.15 (m, 1H), 3.80 – 3.74 (m, 2H), 3.58 – 3.54 (m, 2H), 3.38 (s, 3H), 1.55 (d, J = 6.9 Hz, 3H). ^{13}C{^{1}H} NMR (151 MHz, $CDCl_3$) δ 160.47, 145.20, 142.59, 128.73, 127.54,

126.22, 98.52, 71.56, 68.27, 59.06, 48.73, 21.73. HRMS (ESI) *m/z*, ([M+H]$^+$): calcd. for $C_{14}H_{21}N_2O_4$: 281.1496, found: 281.1495.

(*E*)-*N*-phenethyl-2,5,7-trioxa-8-azadec-8-en-10-amide

^{1}H NMR (600 MHz, CDCl$_3$) δ 7.49 (s, 1H), 7.32 – 7.28 (m, 2H), 7.24 – 7.19 (m, 3H), 6.65 (br. s, 1H), 5.21 (s, 2H), 3.76 – 3.73 (m, 2H), 3.60 – 3.56 (m, 2H), 3.55 – 3.53 (m, 2H), 3.38 (s, 3H), 2.86 (t, *J* = 7.2 Hz, 2H). ^{13}C{^{1}H} NMR (151 MHz, CDCl$_3$) δ 161.33, 144.98, 138.54, 128.75, 128.64, 126.60, 98.52, 71.56, 68.35, 59.05, 40.47, 35.67. HRMS (ESI) *m/z*, ([M+H]$^+$): calcd. for $C_{14}H_{21}N_2O_4$: 281.1496, found: 281.1495.

(*E*)-*N*-(adamantan-1-yl)-2,5,7-trioxa-8-azadec-8-en-10-amide

^{1}H NMR (300 MHz, CDCl$_3$) δ 7.38 (s, 1H), 6.26 (s, 1H,), 5.23 (s, 2H), 3.80 – 3.74 (m, 2H), 3.59 – 3.55 (m, 2H), 3.39 (s, 3H), 2.12 – 2.08 (m, 3H), 2.06 – 2.01 (m, 6H), 1.72 – 1.67 (m, 6H). ^{13}C{^{1}H} NMR (75 MHz, CDCl$_3$) δ 160.18, 146.32, 98.40, 71.58, 68.19, 59.06, 52.12, 41.40, 36.26, 29.39. HRMS (ESI) *m/z*, ([M+H]$^+$): calcd. for $C_{16}H_{27}N_2O_4$: 311.1965, found: 311.1964.

(*E*)-*N*-cyclohexyl-2,5,7-trioxa-8-azadec-8-en-10-amide

^{1}H NMR (300 MHz, CDCl$_3$) δ 7.46 (s,1H), 6.43 (br. s, 1H), 5.23 (s, 2H), 3.86 – 3.73 (m, 3H), 3.59 – 3.52 (m, 2H), 3.39 (s, 3H), 1.98 – 1.89 (m, 2H), 1.78 – 1.68 (m, 2H), 1.66 – 1.57 (m, 1H), 1.43 – 1.28 (m, 2H), 1.26 – 1.13 (m, 3H). ^{13}C{^{1}H} NMR (75 MHz, CDCl$_3$) δ 160.36, 145.45, 98.43, 71.55, 68.20, 59.03, 48.27, 32.92, 25.43, 24.86. HRMS (ESI) *m/z*, ([M+H]$^+$): calcd. for $C_{12}H_{23}N_2O_4$: 259.1652, found: 259.1653.

(*E*)-*N*-(*tert*-butyl)-2,5,7-trioxa-8-azadec-8-en-10-amide

^{1}H NMR (600 MHz, CDCl$_3$) δ 7.39 (s, 1H), 6.39 (br. s, 1H,), 5.23 (s, 2H), 3.78 – 3.75 (m, 2H), 3.58 – 3.54 (m, 2H), 3.38 (s, 3H), 1.39 (s, 9H). ^{13}C{^{1}H} NMR (151 MHz, CDCl$_3$) δ 160.52, 146.21, 98.42, 71.59, 68.19, 59.06, 51.45, 28.67. HRMS (ESI) *m/z*, ([M+H]$^+$): calcd. for $C_{10}H_{21}N_2O_4$: 233.1496, found: 233.1496.

Docking methods:

Biovia Discovery Studio Client v17.2 (Accelrys, San Diego, CA, USA) Dock Ligands protocol (CDOCKER) was used for the docking study with CHARMm force field. CDOCKER is a grid based molecular docking method that employs CHARMm force field.[8,9] The crystal structure of Candida antarctica lipase B (PDB: 5A71) was used as the rigid receptor.[10] The binding site within the lipase B was defined as the largest cavity in the enzyme structure surrounded by a sphere (r = 12.1 Å) to comply with description reported elsewhere (approximately 10 Å x 4 Å wide and 12 Å deep, as measured from Oγ of Ser105 of catalytic triade). Substrate (i.e., carboxylic esters) and amine structures were created and minimized using the MMFF94 force field implemented in ChemBio3D Ultra 13.0 (PerkinElmer, Inc., Waltham, MA, USA).

The following steps were included in the CDOCKER protocol. First, a set of 20 random ligand conformations for each test compound was generated. In the following step, 20 random orientations were kept if the energy was less than the specified threshold value of 300 vdW. This process continued until either a desired number of low-energy orientations were found, or the maximum number of bad orientations had been attempted. The maximum number of bad orientations was set to 800. In the next step each orientation was subjected to simulated annealing molecular dynamics. The temperature was increased to 700 K then cooled to 310 K. The numbers of heating and cooling phase steps during simulated annealing were set to 2000 and 5000, respectively. For the simulated annealing refinement, grid extension (8.0 Å) was used. In the subsequent step, a final minimization of each refined pose of the ligand in the rigid receptor is performed using full potential. In the end, for each final pose, the CHARMm energy (interaction energy plus ligand strain) and the interaction energy alone are calculated. The poses are sorted by CHARMm energy and the 30 top scored (most negative, thus favorable for binding) poses are retained.

The representative pose of each of the docked ligands was chosen based on the highest consensus score across calculated scoring functions estimating binding affinities, implemented in the Biovia Discovery Studio Client's Score Ligand Poses protocol. The following scoring functions were calculated: CDOCKER Scores, LigScore1, LigScore2, PLP1, PLP2, Jain, PMF, PMF04, Ludi Energy Estimate 1, Ludi Energy Estimate 2, and Ludi Energy Estimate 3. To identify the poses of docked ligands that score high in more than one scoring function, the Biovia Discovery Studio Client's Consensus Score protocol was used that calculates the consensus scores of a series of docked ligands for which other scores have been previously computed. For each selected scoring function, the ligands are listed by score in descending order. The consensus score for a ligand is an integer between zero (none of the scores are in the top-ranking percentile of receptor-ligand poses) and the total number of scores (all of the scores are in the top-ranking percentile) listed in Input Properties. Thus, the following scoring functions were calculated for each obtained receptor-ligand pose and their scores used to calculate the consensus score: -CDOCKER_ENERGY, -CDOCKER_INTERACTION_ENERGY, LigScore1, LigScore2, PLP1, PLP2, Jain, PMF, PMF04, Ludi Energy Estimate 1, Ludi Energy Estimate 2, and Ludi Energy Estimate 3. Consensus Percentage was set to 10 to specify the percentage of top receptor-ligand poses to include in the consensus. Use Best Pose only was set to False. Finally, the poses with highest consensus score were chosen as the representative poses of each of the docked ligands and their receptor-ligand model complexes analyzed for non-bonding interactions.

The selected poses of docked ligands were minimized using Biovia Discovery Studio Client v17.2 protocol Minimization. Smart Minimizer algorithm was used to perform the minimization. The applied algorithm performs 1000 steps of Steepest Decent with a RMS gradient tolerance of 3, followed by Conjugate Gradient minimization, with the values of Max Steps and RMS Gradient set to 1000 and

0.01, respectively. Generalized Born with Molecular Volume (GBMV) implicit solvent model[11] was used in the calculation with the effective Born radii calculated by numerical integration of molecular volume.[12] The non-polar surface area was used to approximate the non-polar component of the solvation energy. Implicit solvent dielectric constant was set to 80. Distance cutoff value used for counting non-bonded interaction pairs was set to 14.0 Å.

NMR spectra:

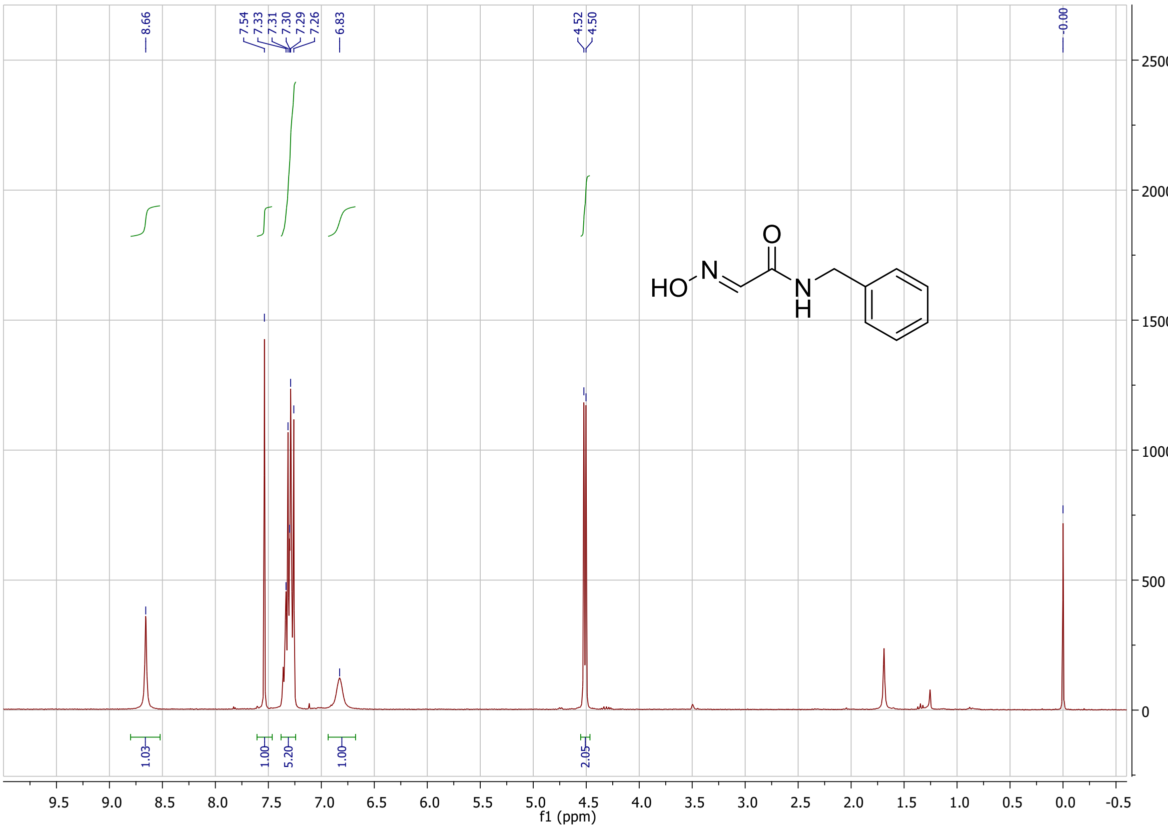

8.66
7.54
7.33
7.31
7.30
7.29
7.26
6.83
4.52
4.50
0.00
1.03
1.00
5.20
1.00
2.05
HO
N
O
N
H
f1 (ppm)

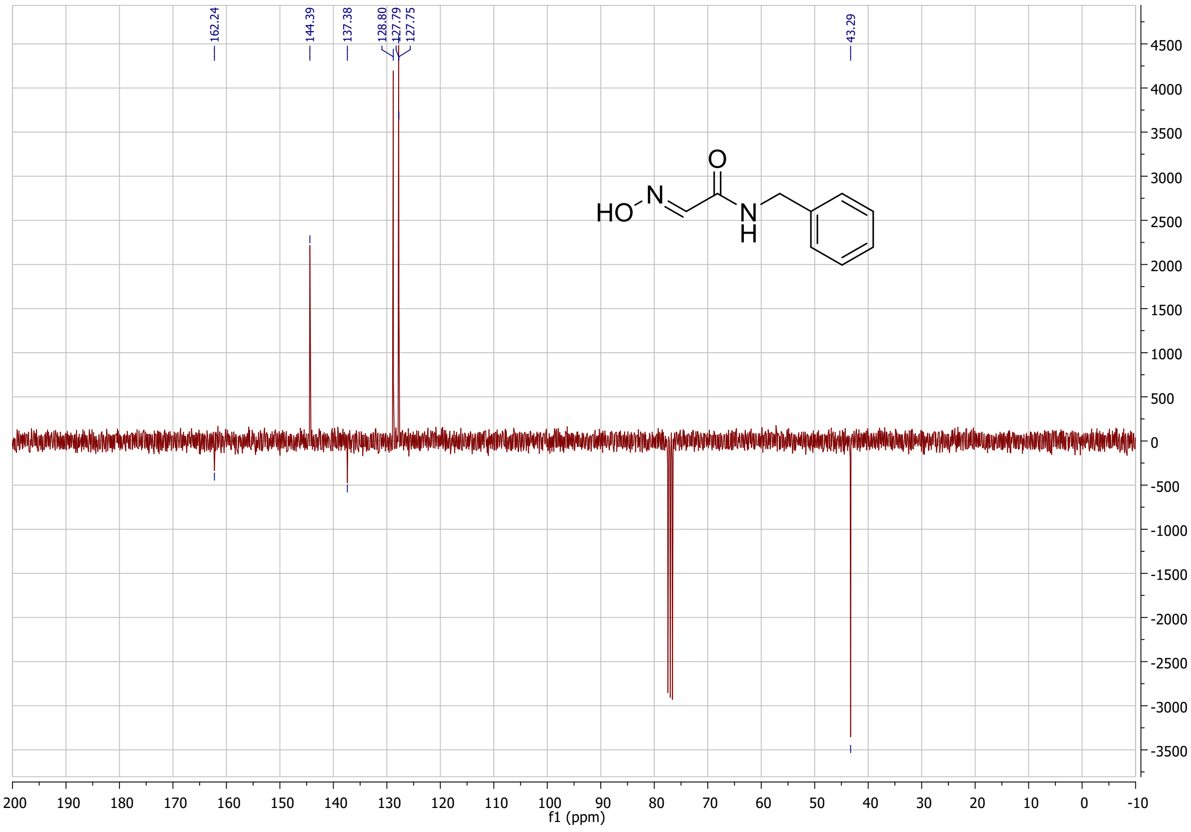

162.24
144.39
137.38
128.80
127.79
127.75
43.29
HO
N
O
N
H
f1 (ppm)

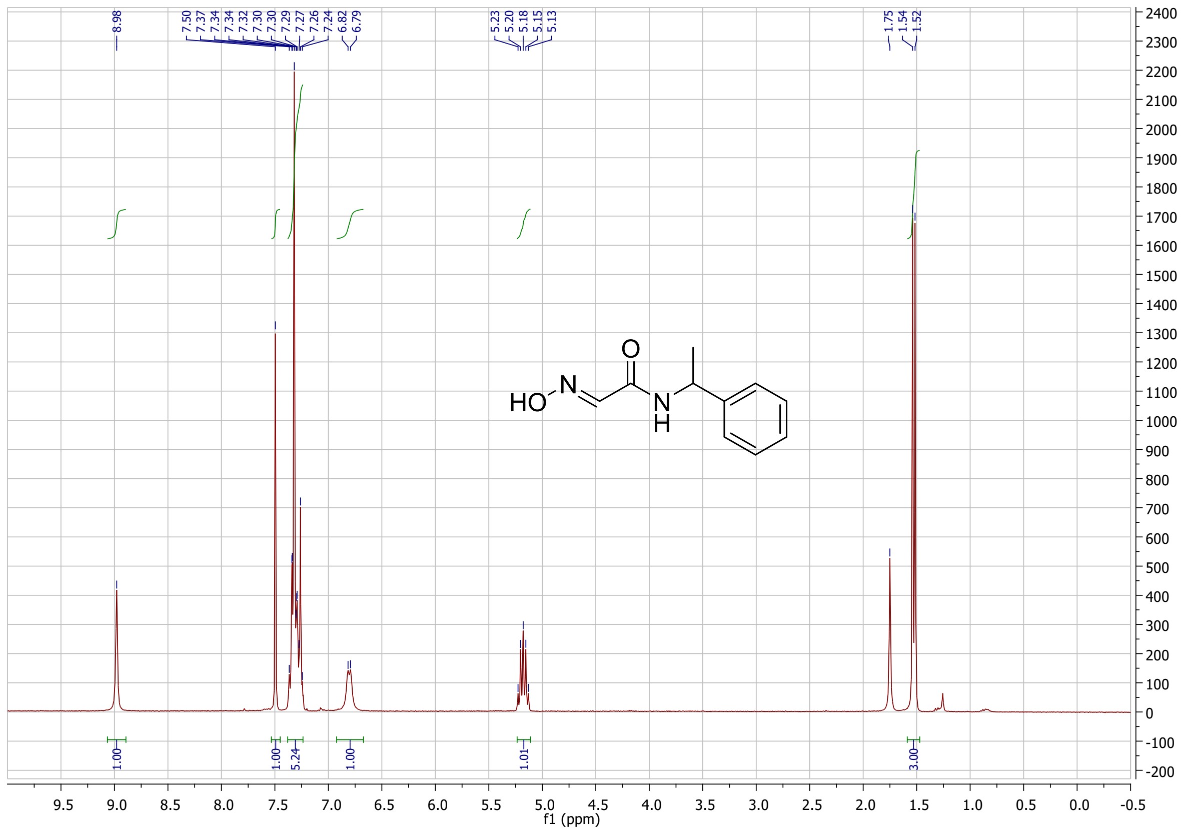
8.98
7.50
7.37
7.34
7.34
7.32
7.30
7.30
7.29
7.27
7.26
7.24
6.82
6.79
5.23
5.20
5.18
5.15
5.13
1.75
1.54
1.52
HO
N
O
N
H
1.00
1.00
5.24
1.00
1.01
3.00
f1 (ppm)

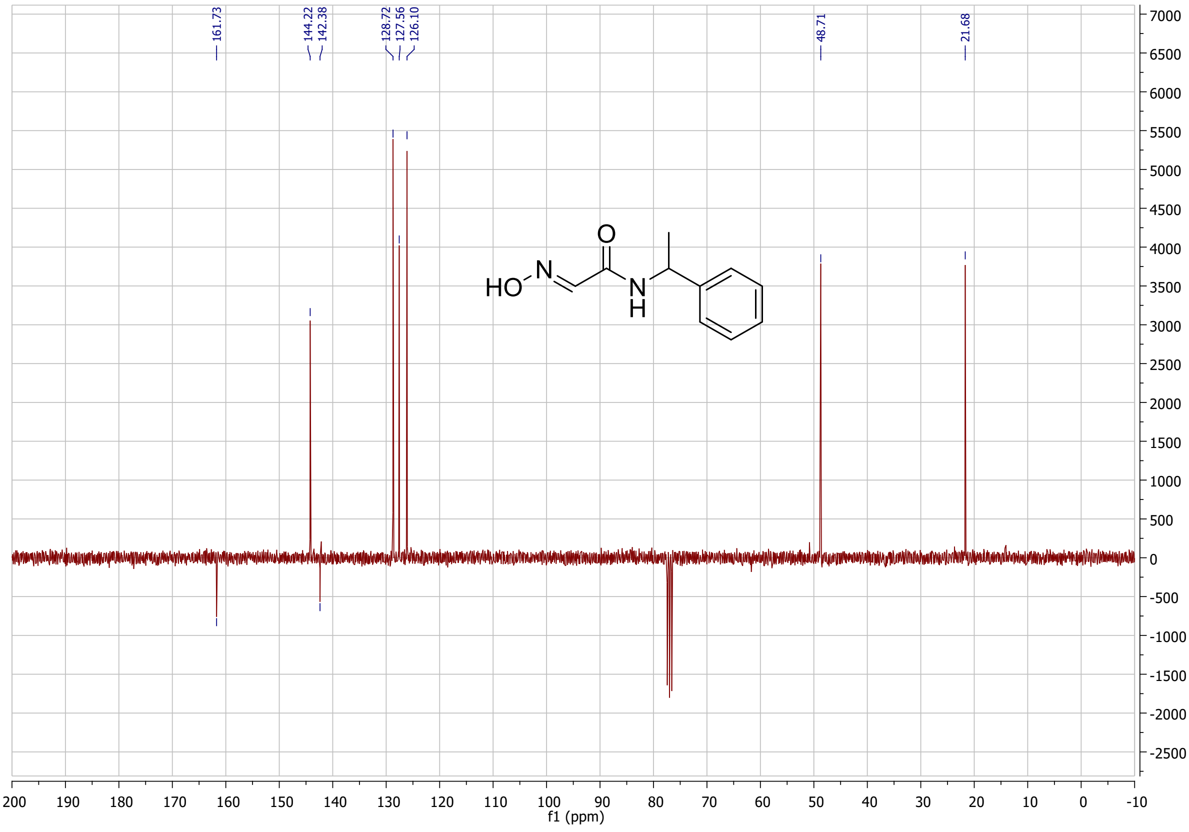
161.73
144.22
142.38
128.72
127.56
126.10
48.71
21.68
HO
N
O
N
H
f1 (ppm)

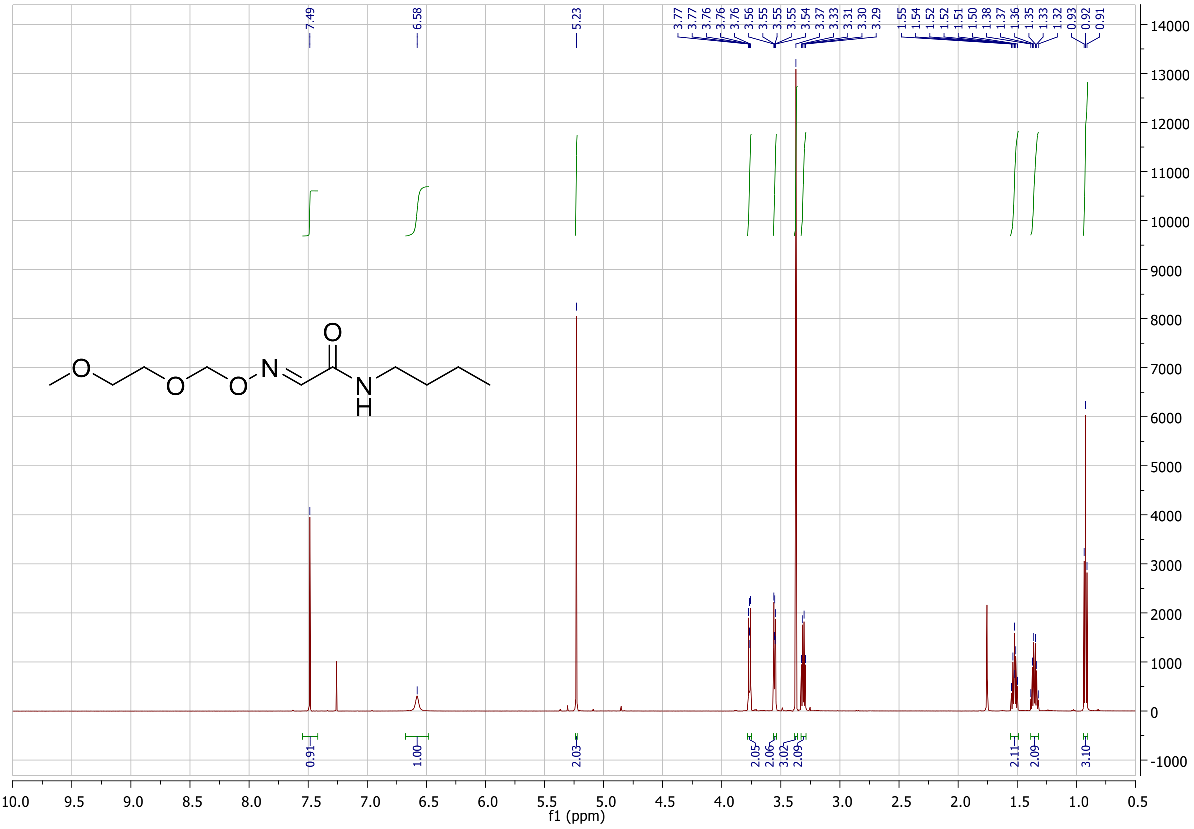

f1 (ppm)

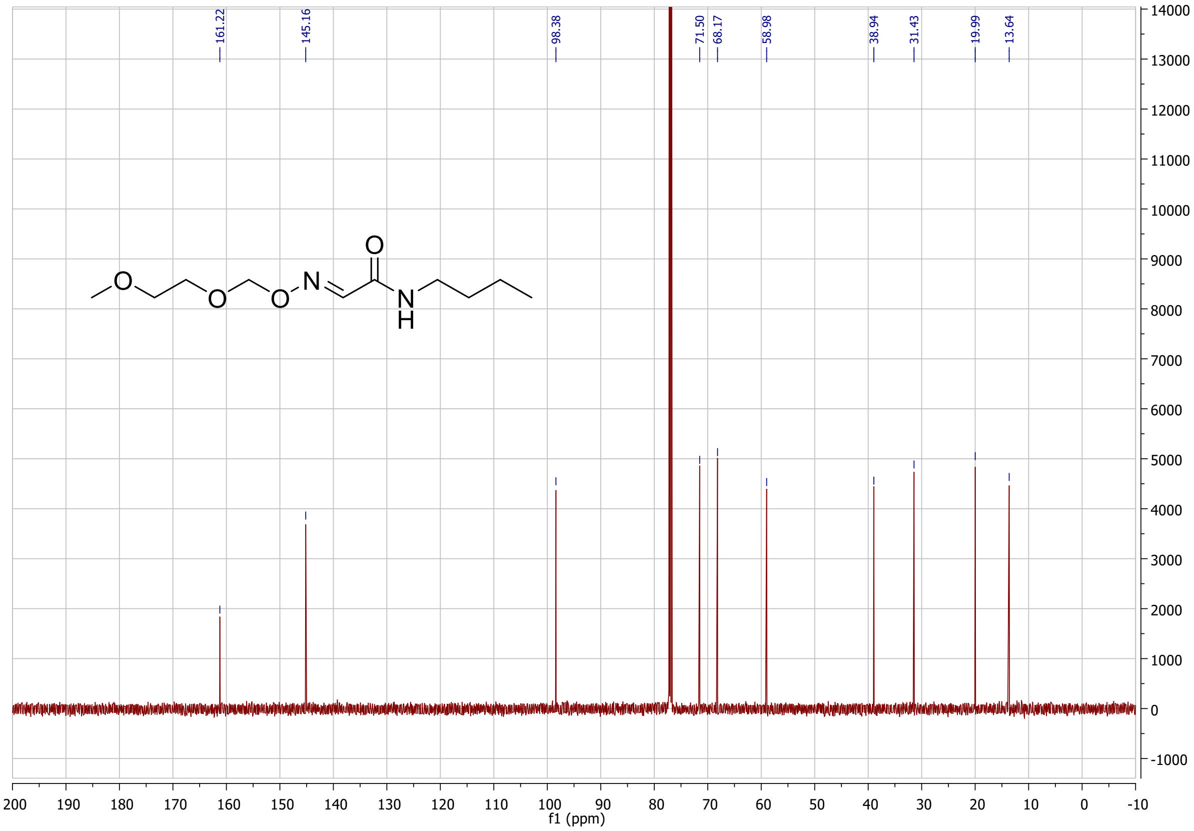

161.22
145.16
98.38
71.50
68.17
58.98
38.94
31.43
19.99
13.64
f1 (ppm)

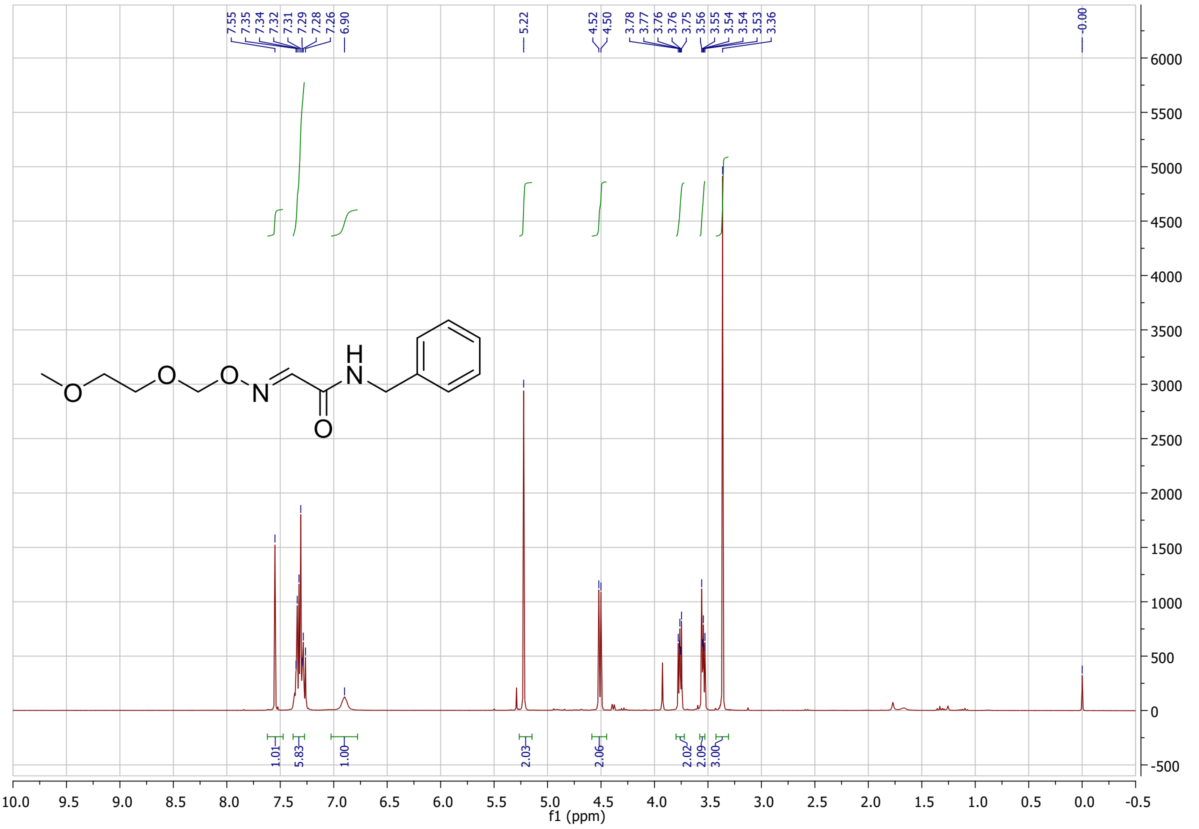
7.55
7.35
7.34
7.32
7.31
7.29
7.28
7.26
6.90
5.22
4.52
4.50
3.78
3.77
3.76
3.76
3.75
3.56
3.55
3.54
3.54
3.53
3.36
-0.00
1.01
5.83
1.00
2.03
2.06
2.02
2.09
3.00
f1 (ppm)
H
N
O
O
O
N
O

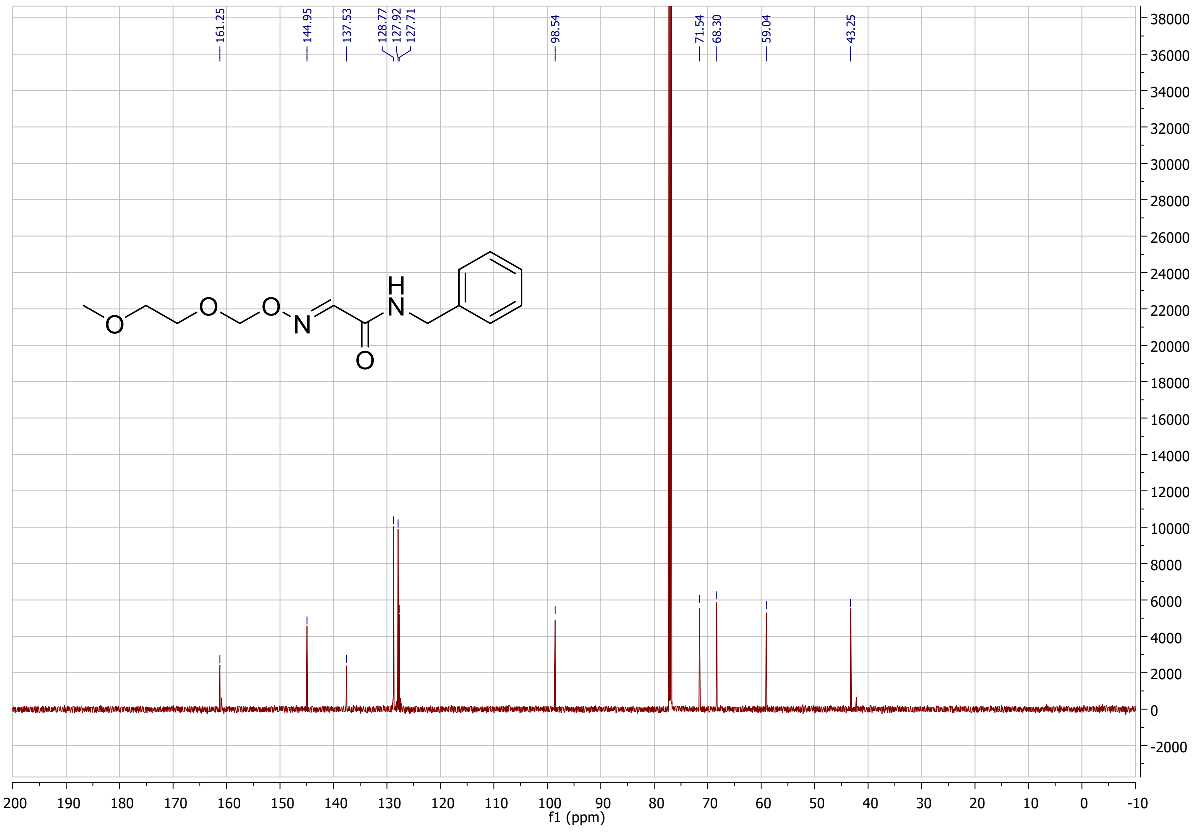
161.25
144.95
137.53
128.77
127.92
127.71
98.54
71.54
68.30
59.04
43.25
f1 (ppm)
H
N
O
O
O
N
O

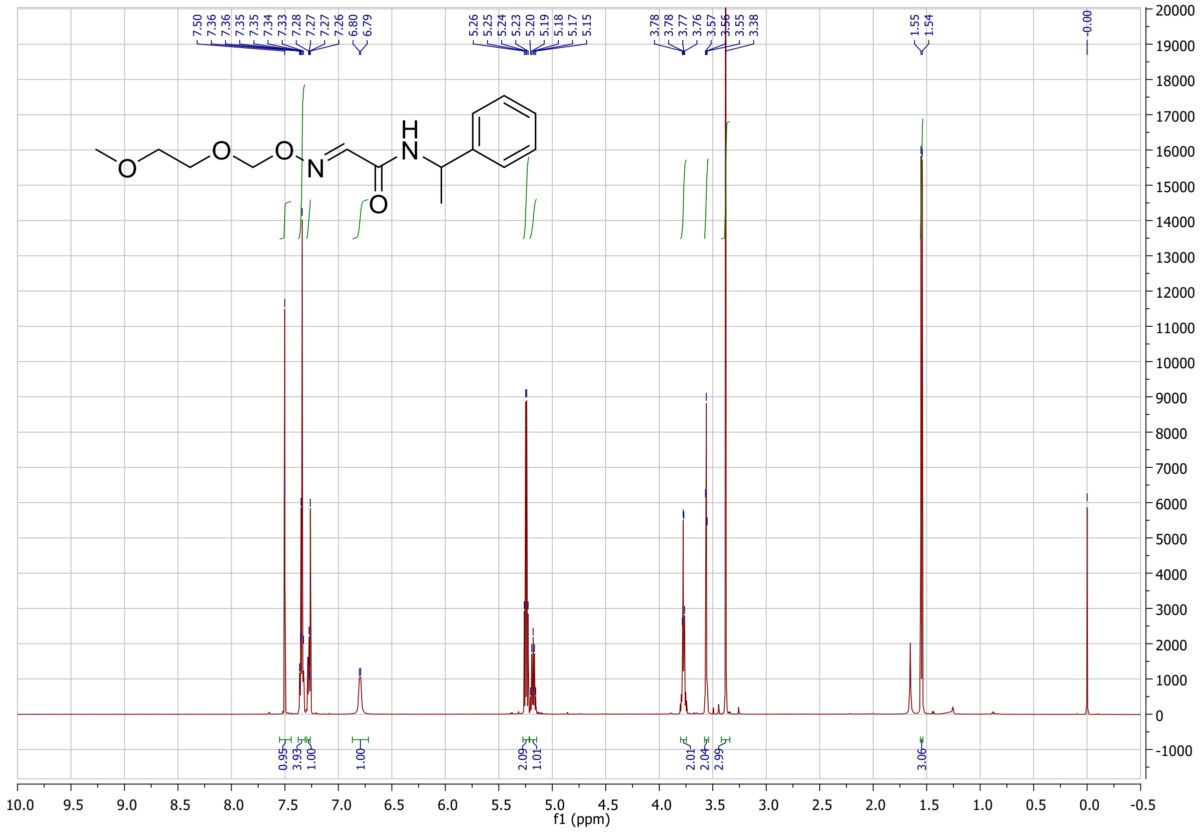
7.50
7.36
7.36
7.35
7.35
7.34
7.33
7.28
7.27
7.27
7.26
6.80
6.79
5.26
5.25
5.24
5.23
5.20
5.19
5.18
5.17
5.15
3.78
3.78
3.77
3.76
3.57
3.56
3.55
3.38
1.55
1.54
-0.00
0.95
3.93
1.00
1.00
2.09
1.01
2.01
2.04
2.99
3.06
f1 (ppm)

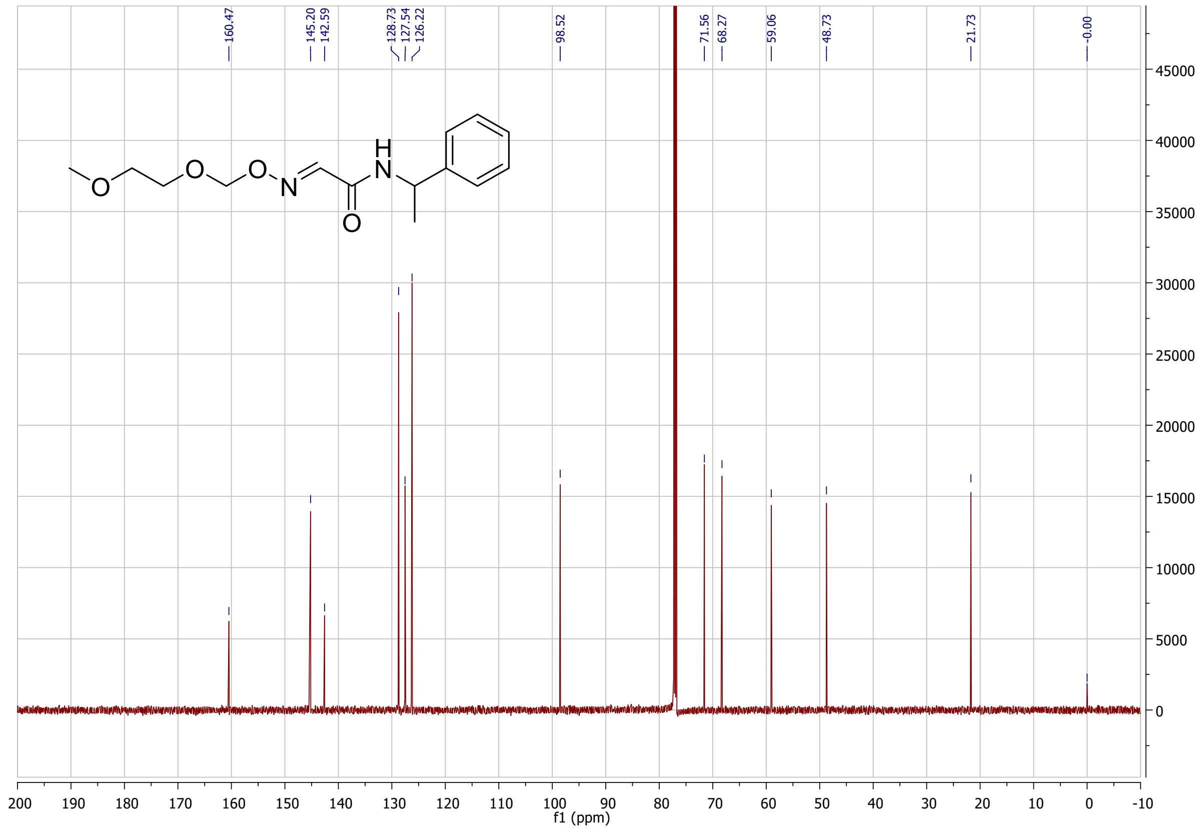
160.47
145.20
142.59
128.73
127.54
126.22
98.52
71.56
68.27
59.06
48.73
21.73
-0.00
f1 (ppm)

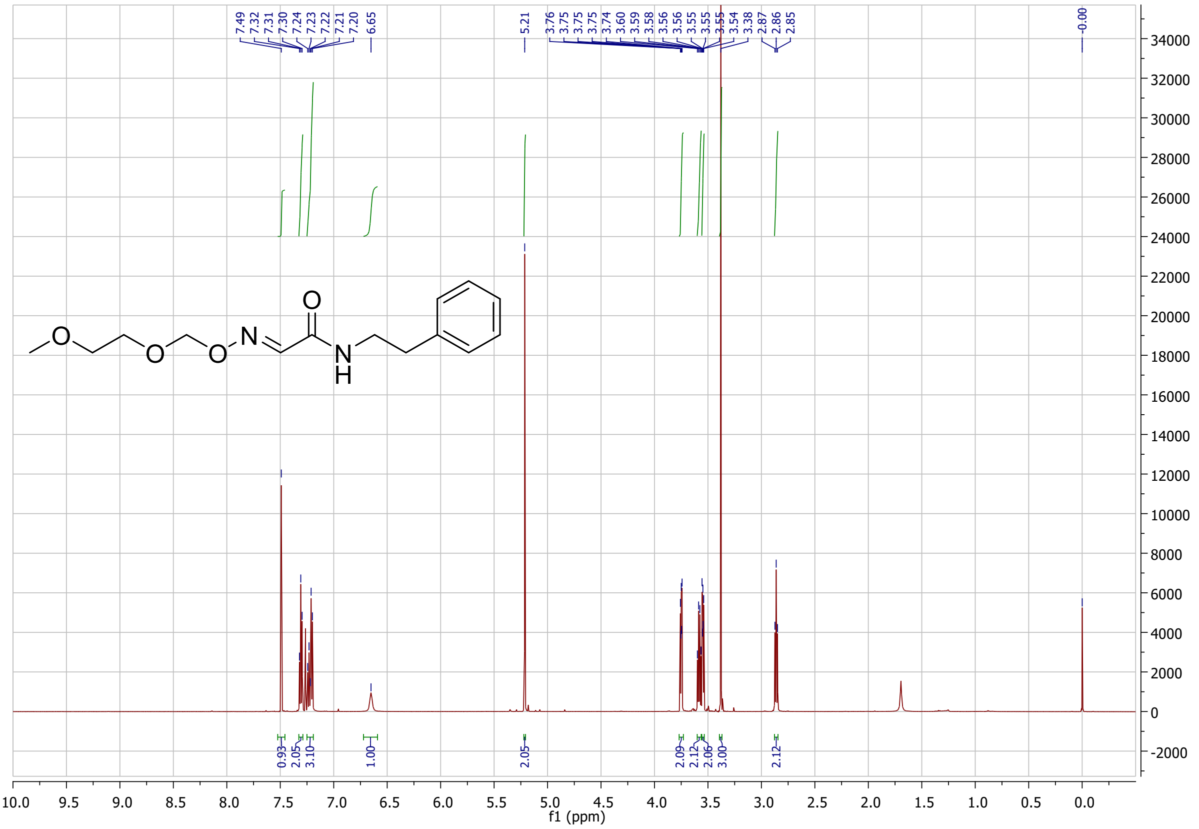
7.49
7.32
7.31
7.30
7.24
7.23
7.22
7.21
7.20
6.65
5.21
3.76
3.75
3.75
3.75
3.74
3.60
3.59
3.58
3.56
3.56
3.55
3.55
3.55
3.54
3.38
2.87
2.86
2.85
0.00
0.93
2.05
3.10
1.00
2.05
2.09
2.12
2.06
3.00
2.12
f1 (ppm)

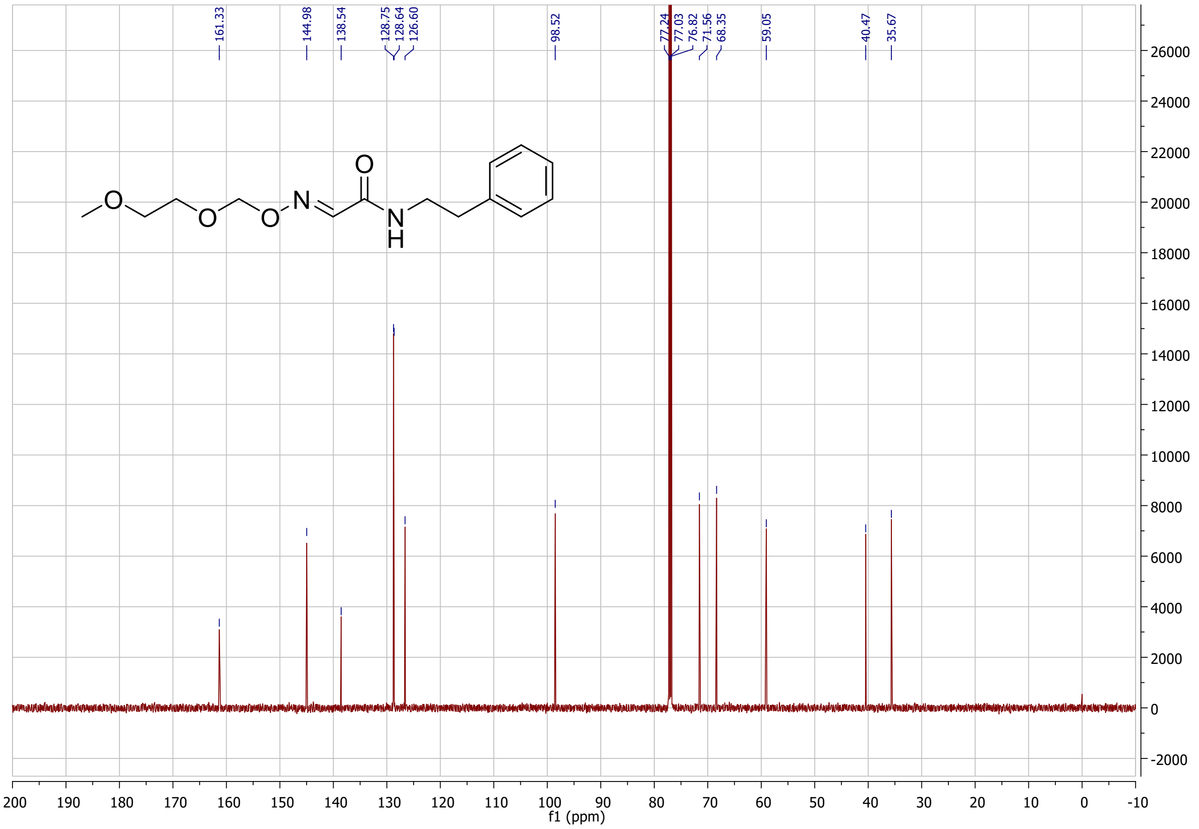
161.33
144.98
138.54
128.75
128.64
126.60
98.52
77.24
77.03
76.82
71.56
68.35
59.05
40.47
35.67
f1 (ppm)

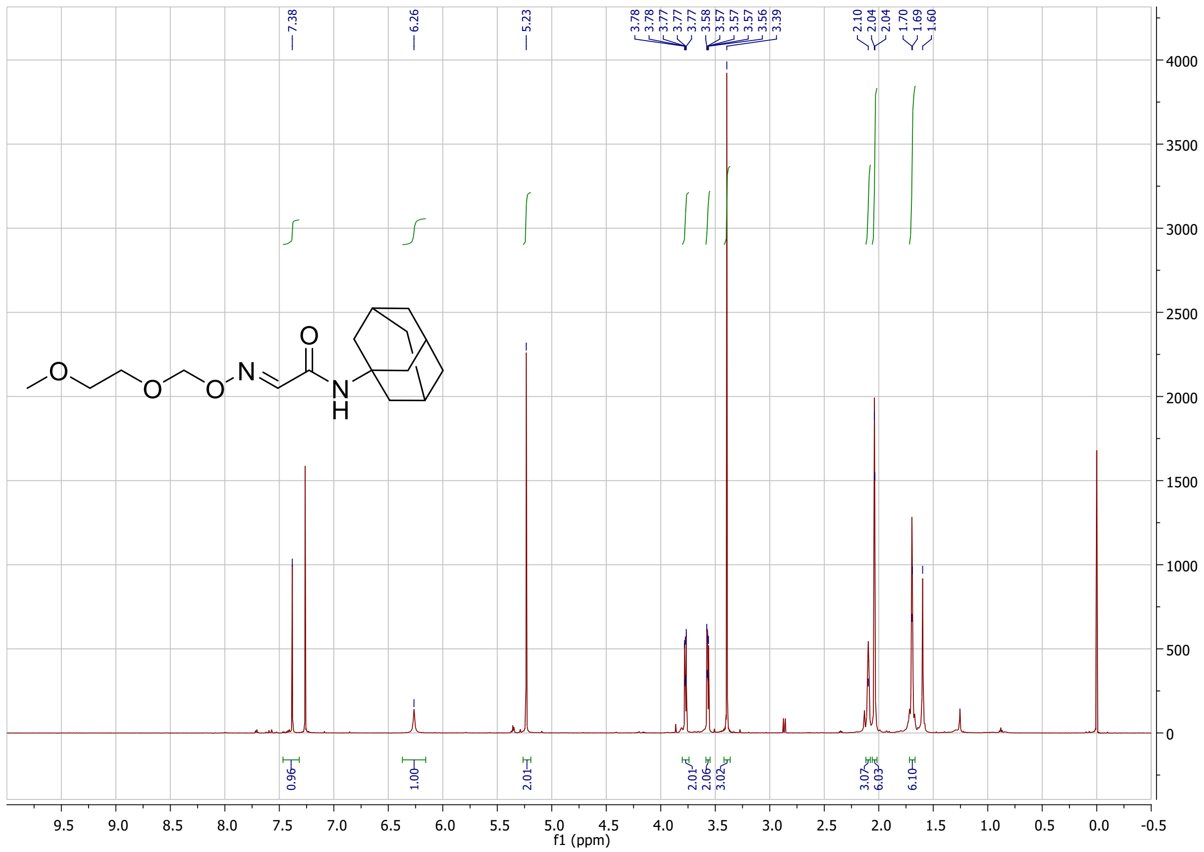

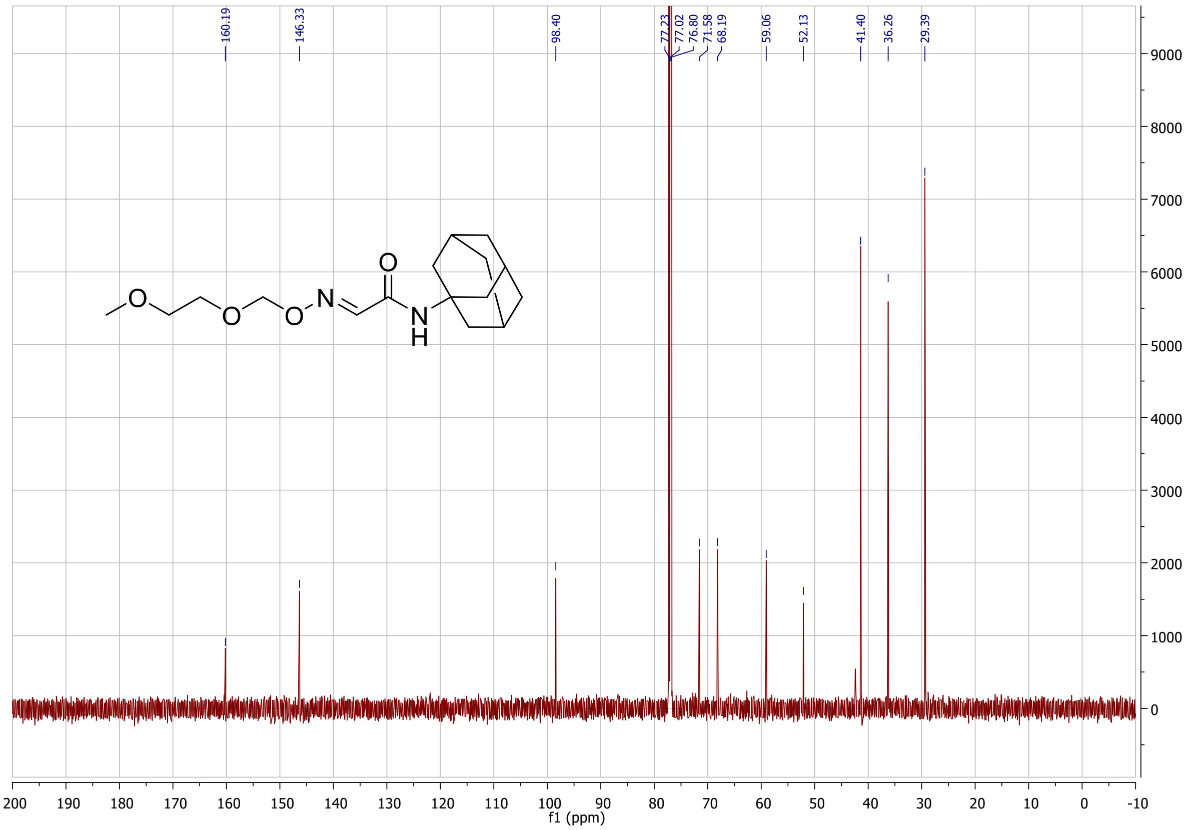

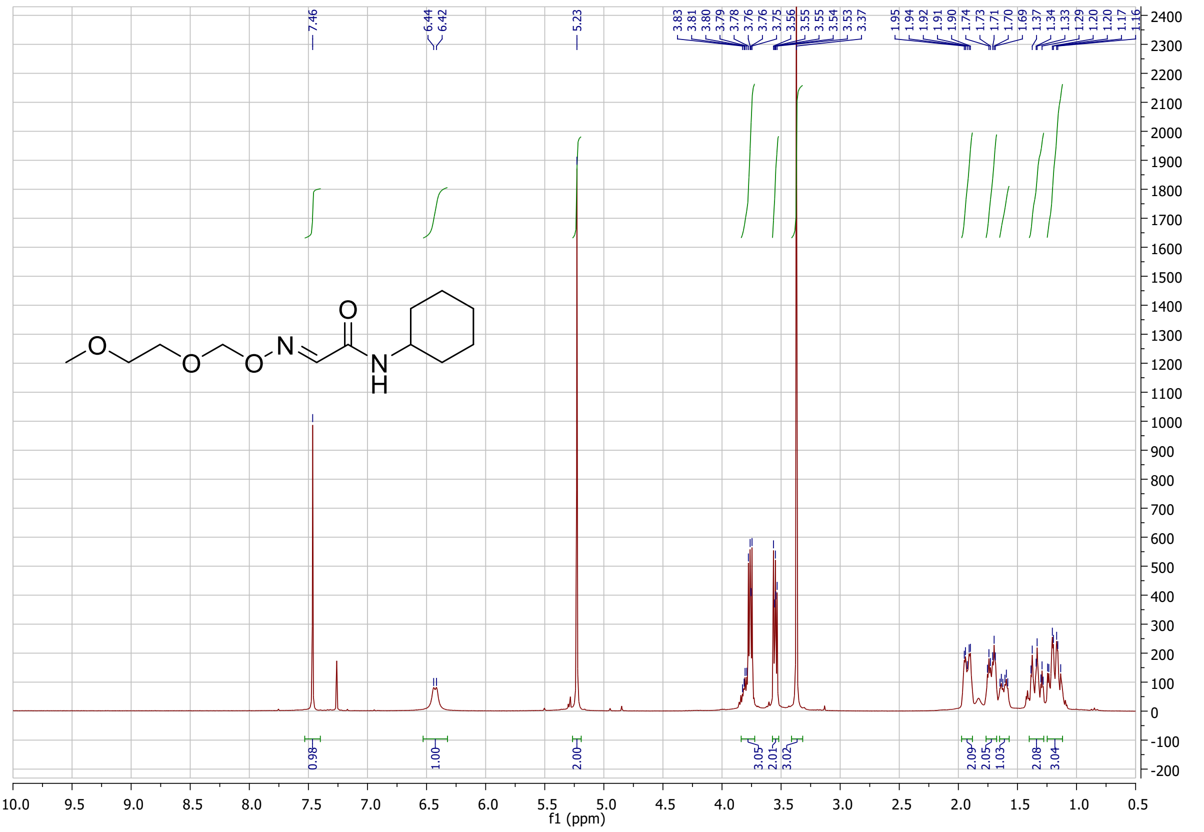
f1 (ppm)
O
N
H

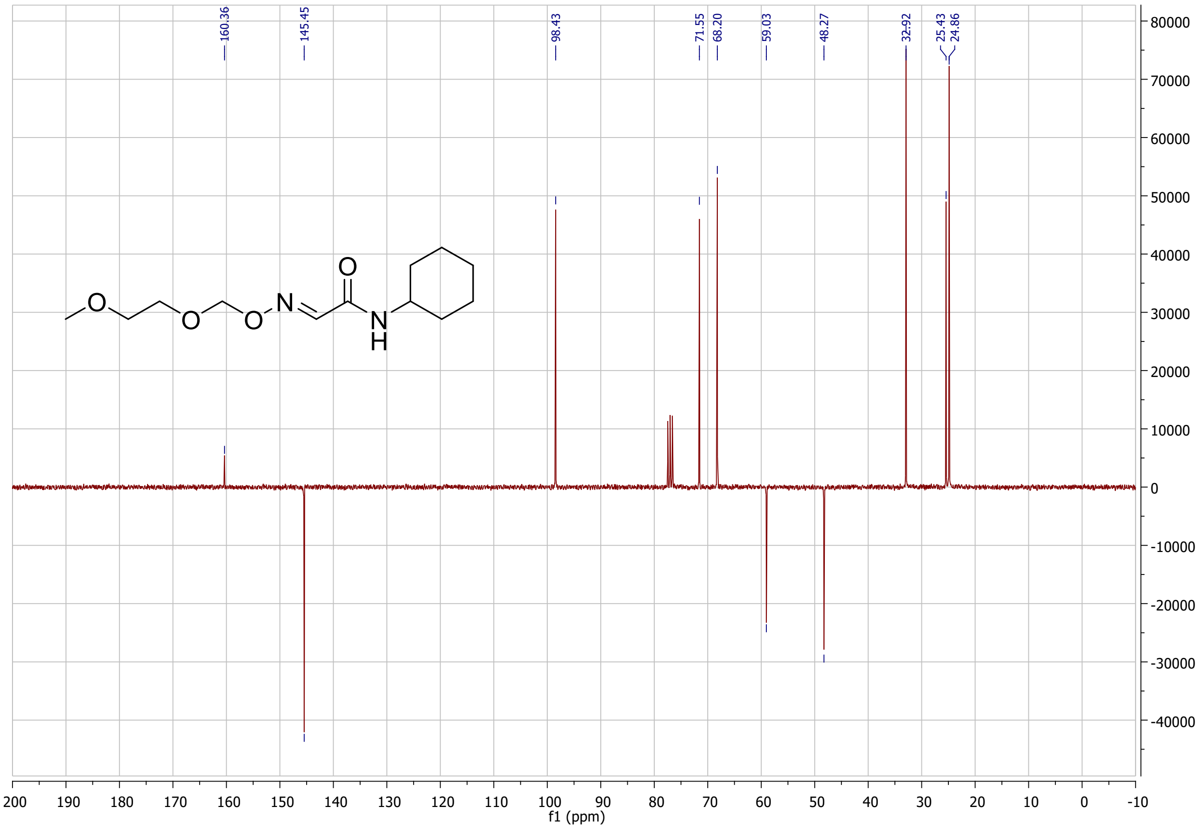
f1 (ppm)
O
N
H

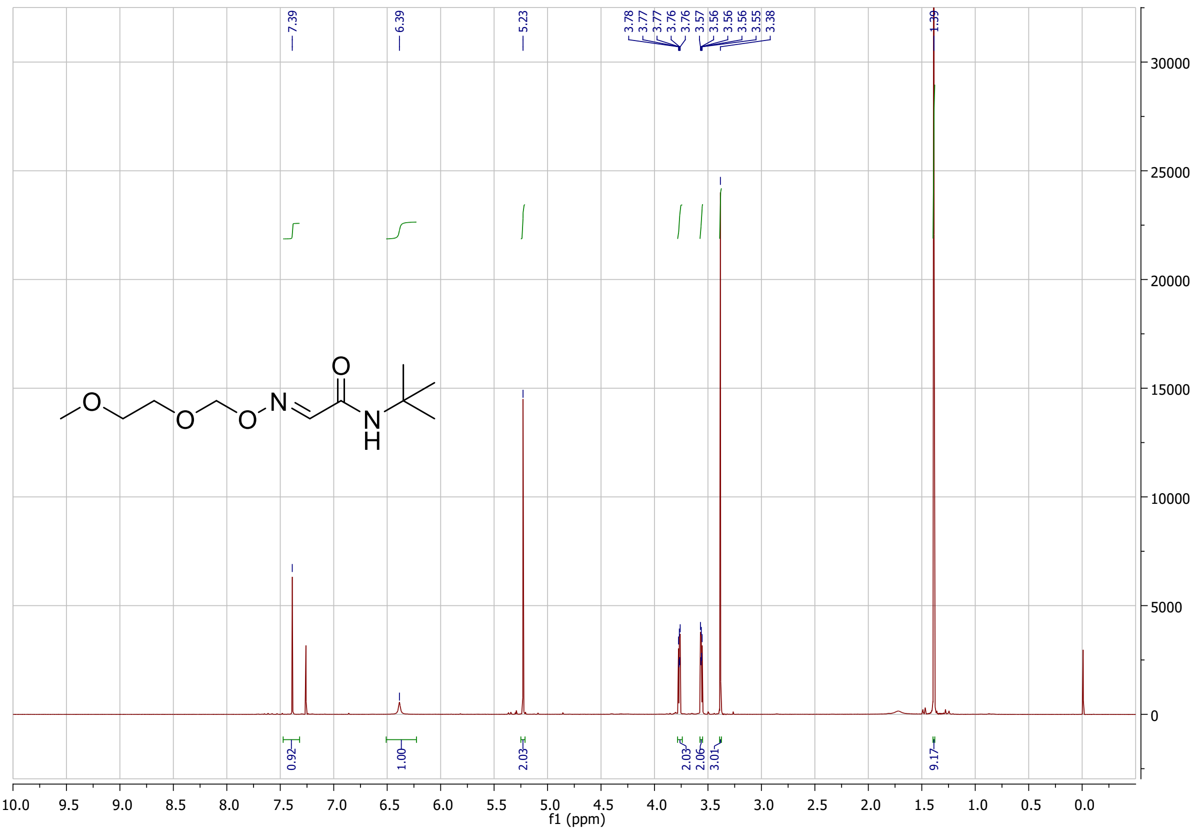
7.39
6.39
5.23
3.78 3.77 3.77 3.76 3.76 3.57 3.56 3.56 3.56 3.55 3.38
1.39
0.92
1.00
2.03
2.03
2.06
3.01
9.17
10.0 9.5 9.0 8.5 8.0 7.5 7.0 6.5 6.0 5.5 5.0 4.5 4.0 3.5 3.0 2.5 2.0 1.5 1.0 0.5 0.0
f1 (ppm)
30000 25000 20000 15000 10000 5000 0

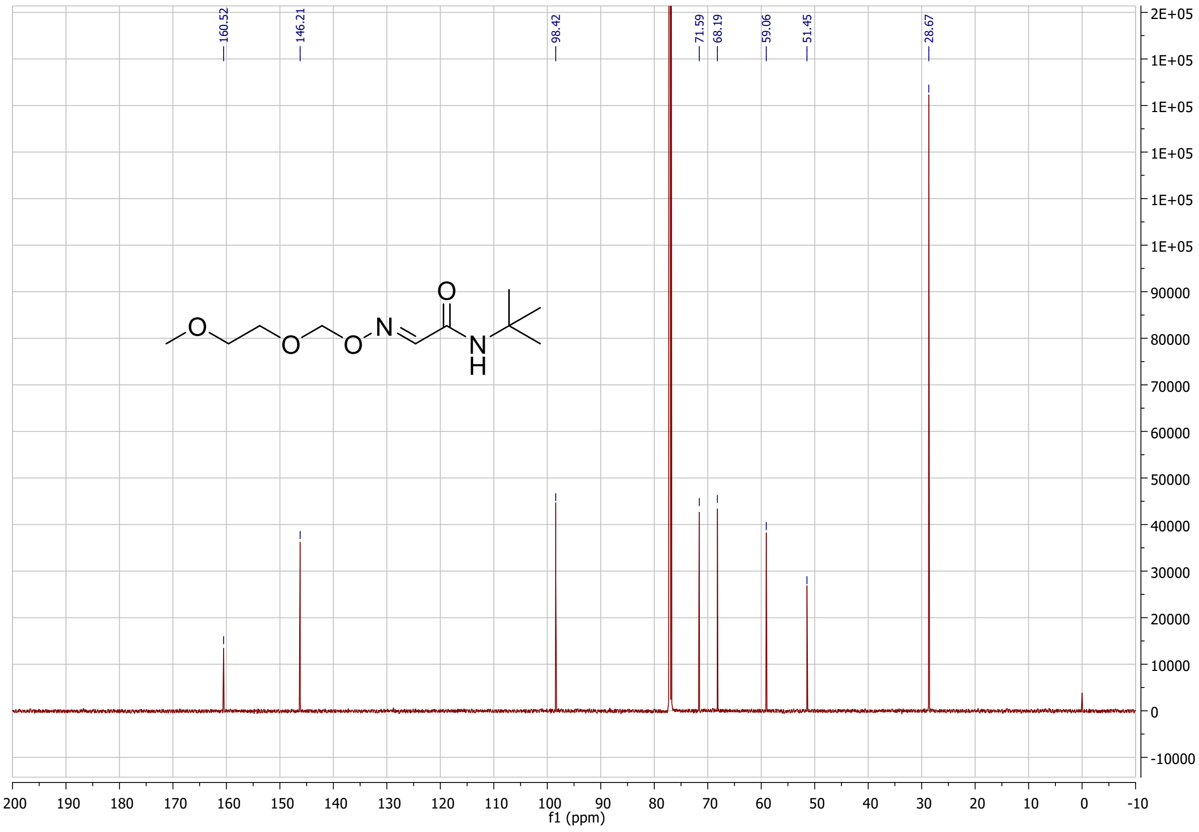
160.52
146.21
98.42
71.59
68.19
59.06
51.45
28.67
200 190 180 170 160 150 140 130 120 110 100 90 80 70 60 50 40 30 20 10 0 -10
f1 (ppm)
2E+05 1E+05 1E+05 1E+05 1E+05 1E+05 90000 80000 70000 60000 50000 40000 30000 20000 10000 0 -10000

HRMS analysis

(*E*)-*N*-benzyl-2-(hydroxyimino)acetamide

Calculated $[M+H]^+$: 179,0815

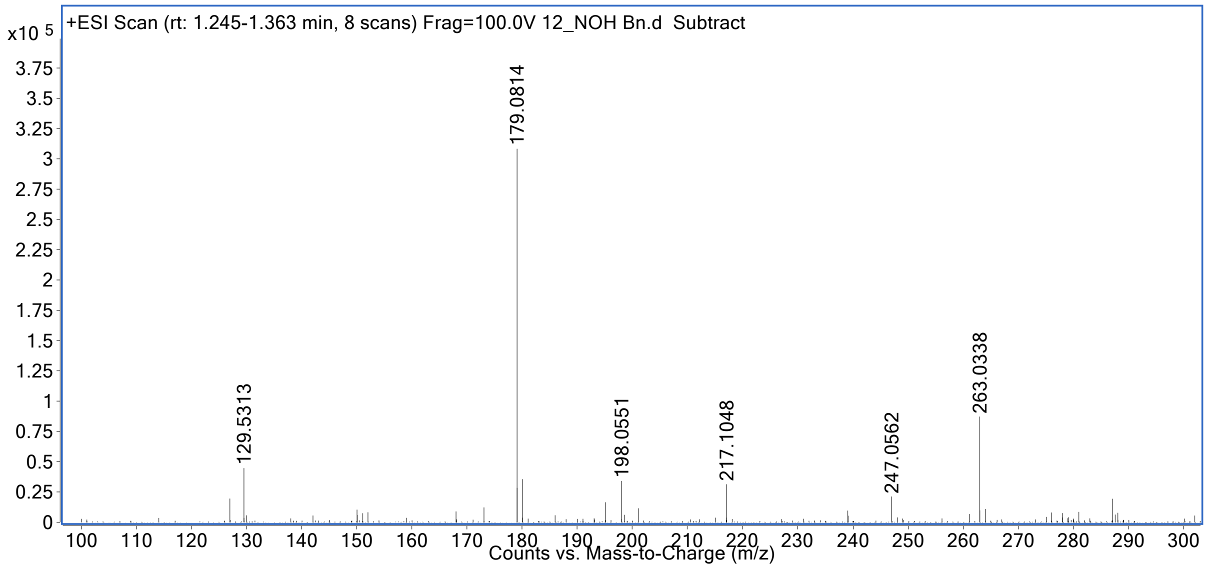

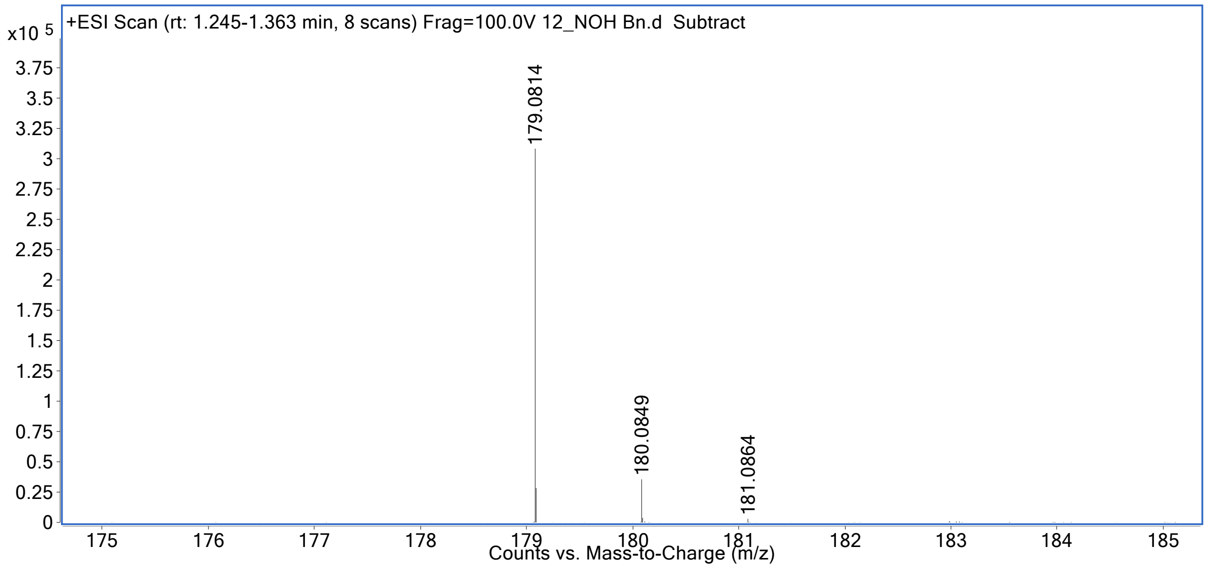

(*E*)-2-(hydroxyimino)-*N*-(1-phenylethyl)acetamide

Calculated $[M+H]^+$: 193,0972

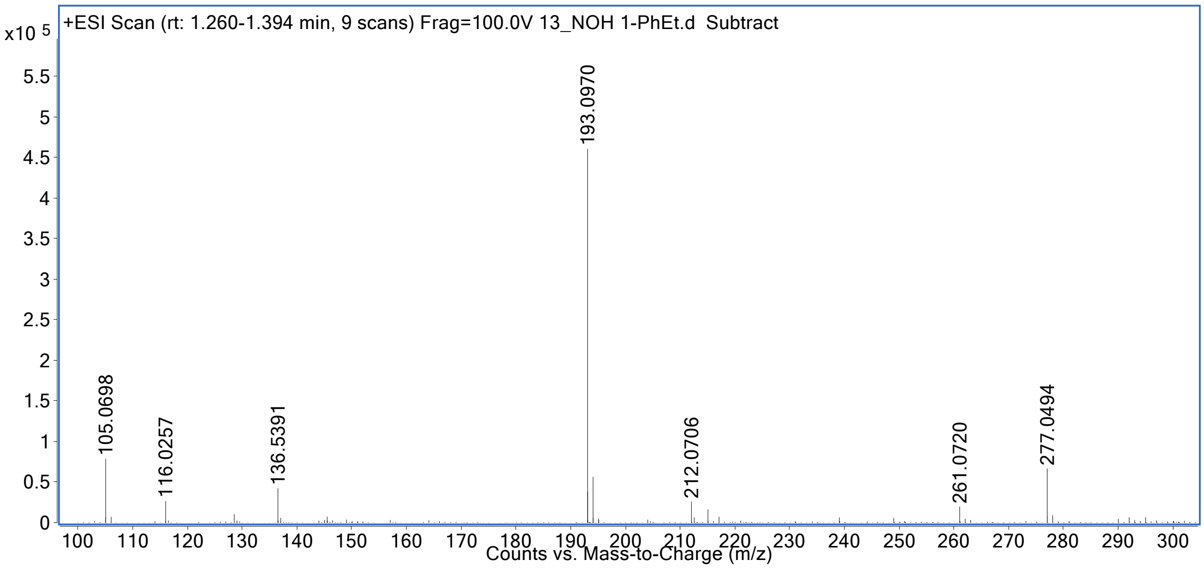

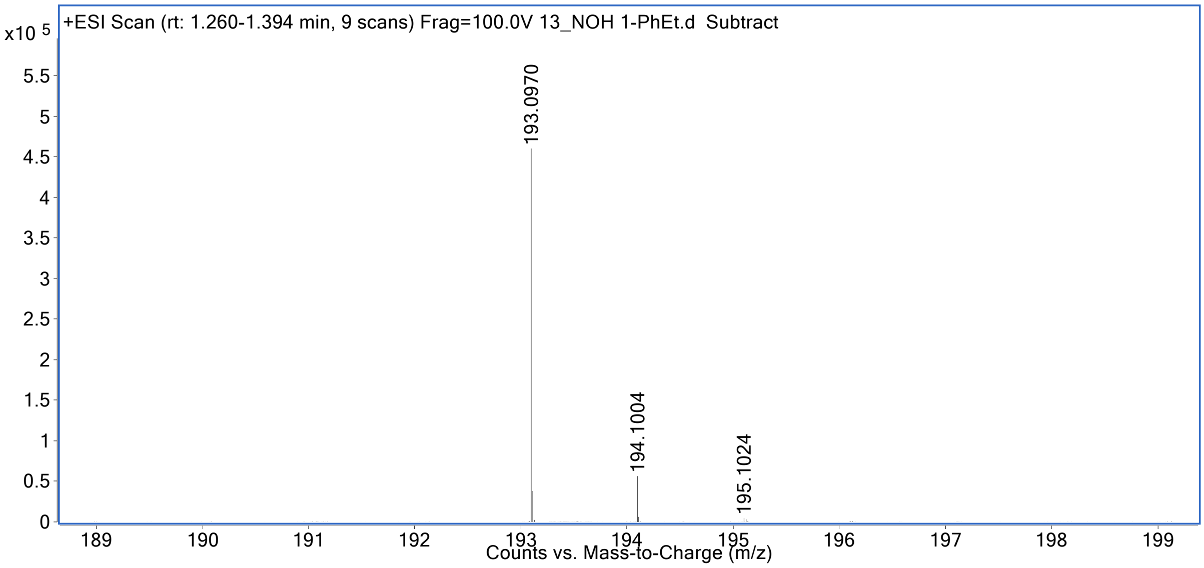

<u>(*E*)-*N*-butyl-2,5,7-trioxa-8-azadec-8-en-10-amide</u>

Calculated $[M+H]^+$: 233,1496

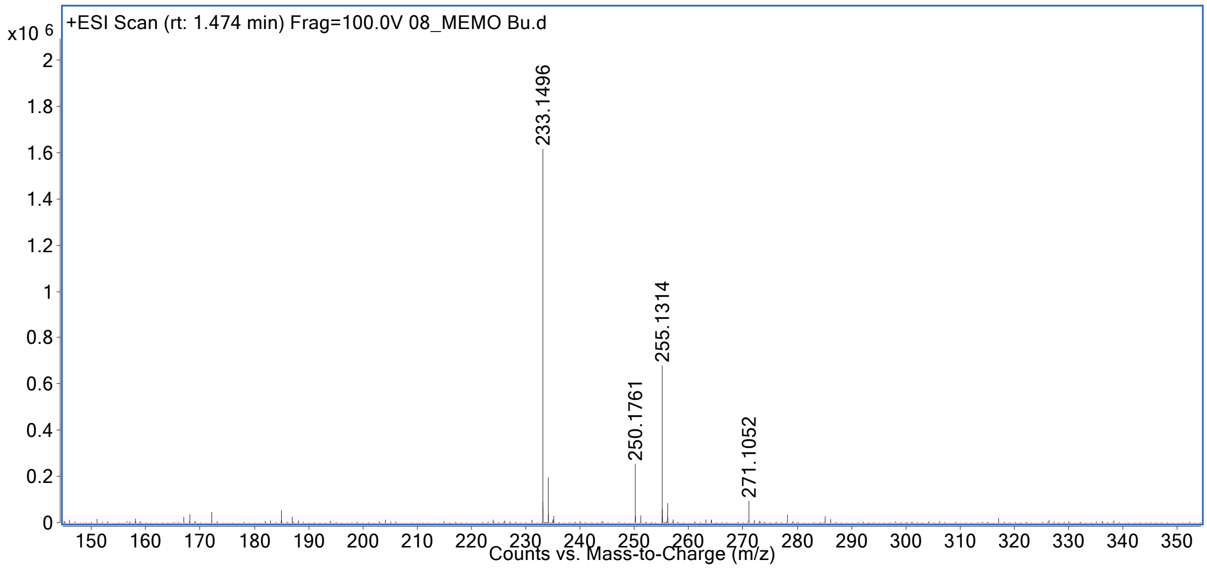

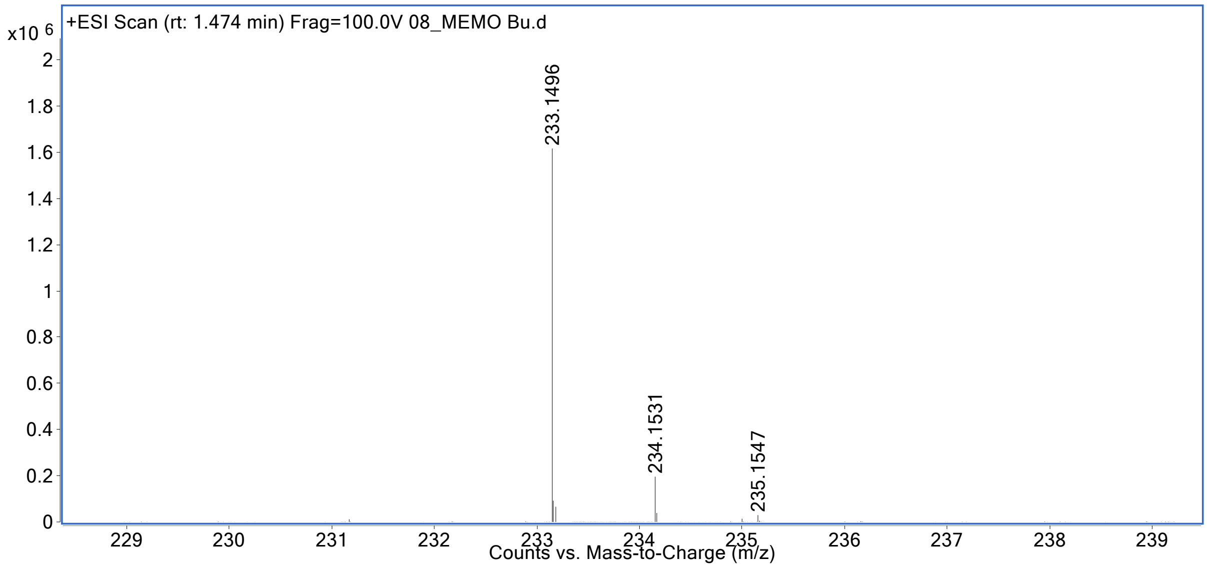

(*E*)-*N*-benzyl-2,5,7-trioxa-8-azadec-8-en-10-amide

Calculated $[M+H]^+$: 267,1339

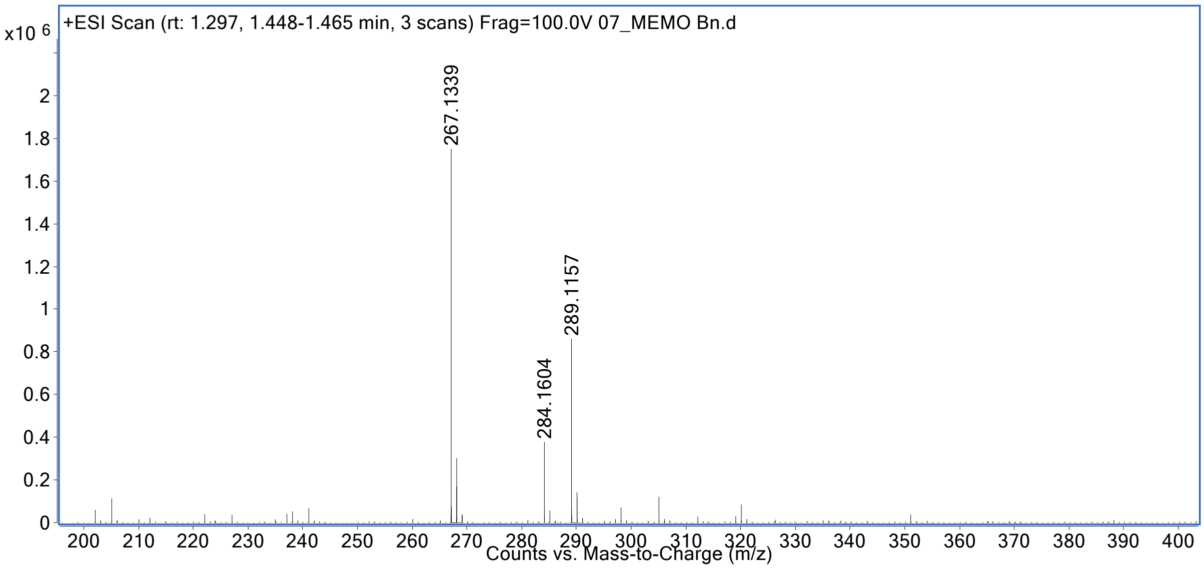

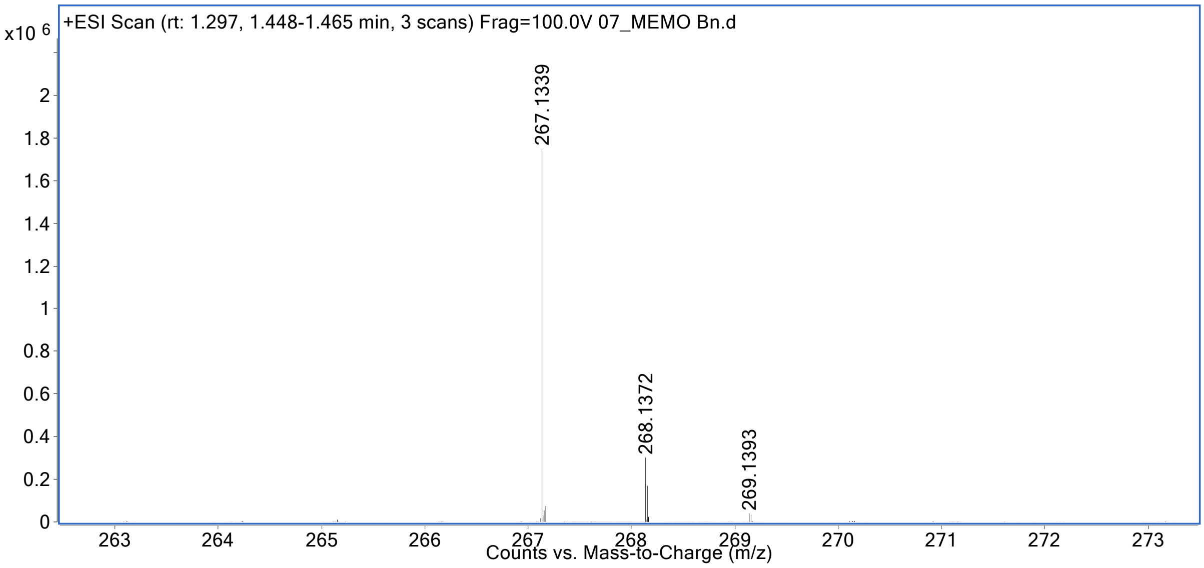

(*E*)-*N*-(1-phenylethyl)-2,5,7-trioxa-8-azadec-8-en-10-amide

Calculated $[M+H]^+$: 281,1496

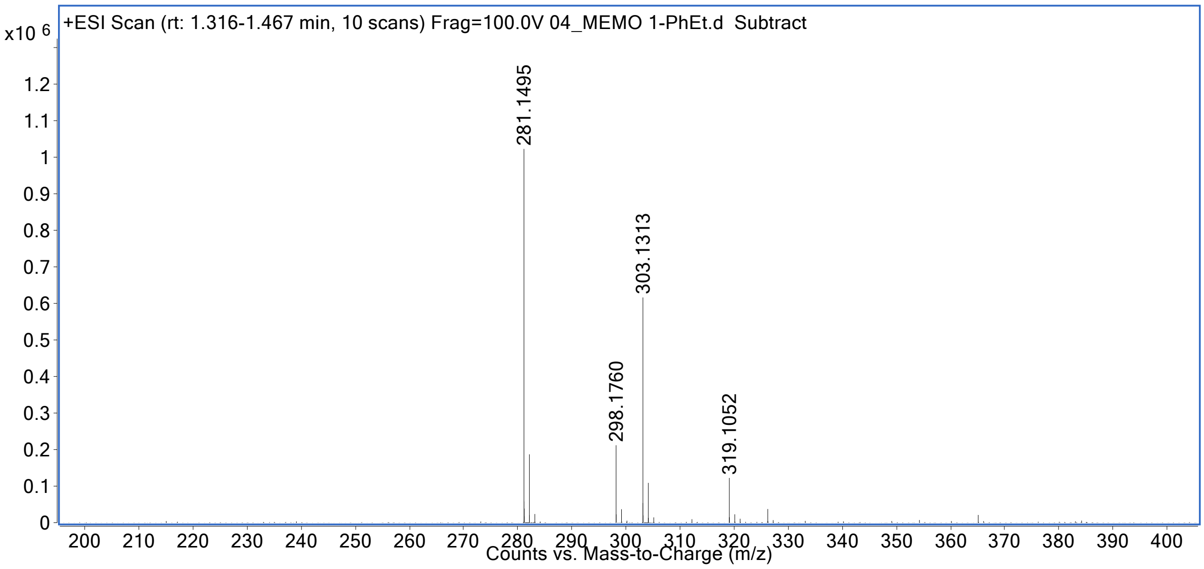

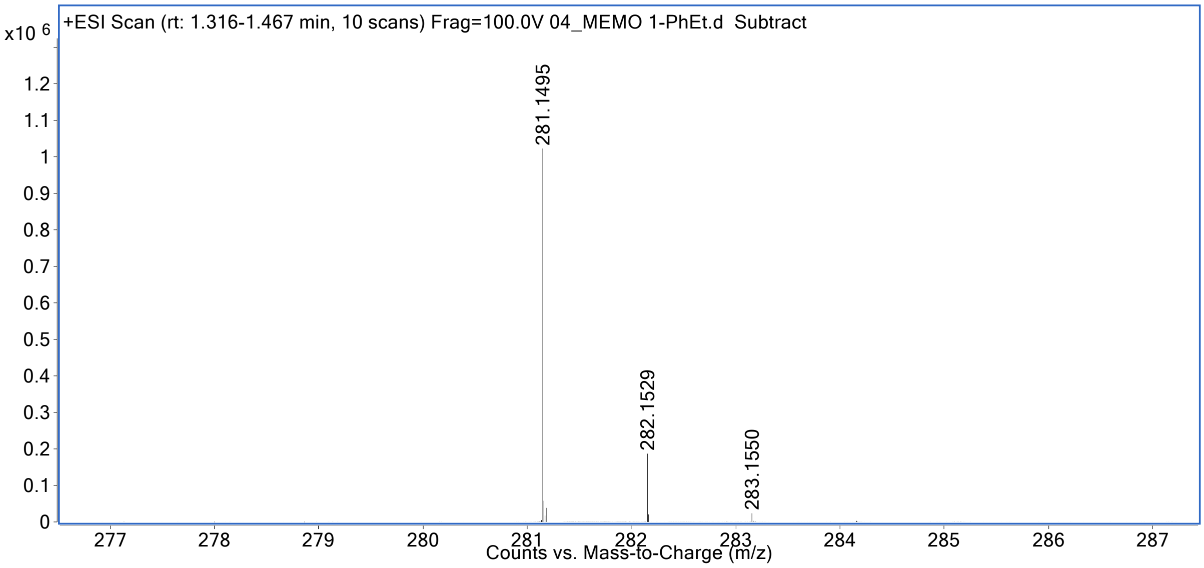

<u>(*E*)-*N*-phenethyl-2,5,7-trioxa-8-azadec-8-en-10-amide</u>

Calculated $[M+H]^+$: 281,1496

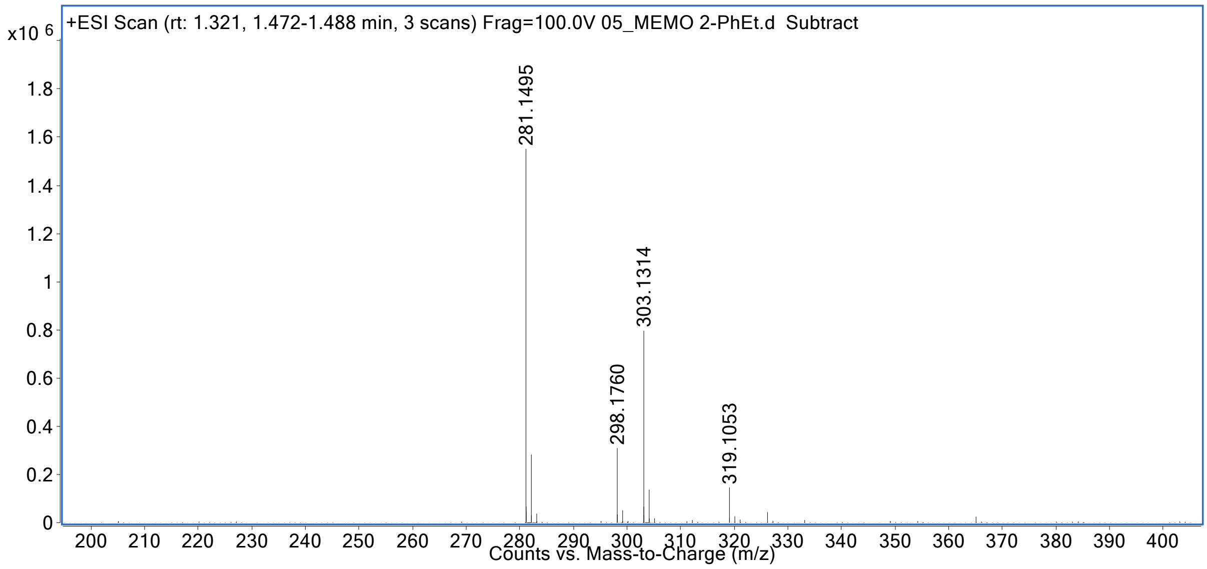

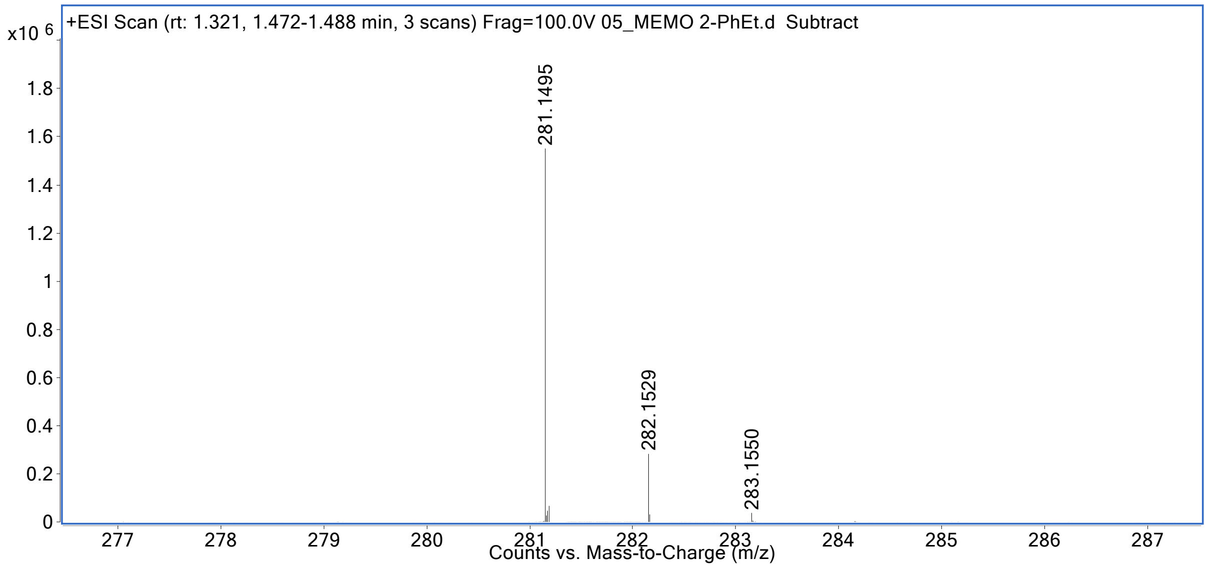

(*E*)-*N*-(adamantan-1-yl)-2,5,7-trioxa-8-azadec-8-en-10-amide

Calculated $[M+H]^+$: 311,1965

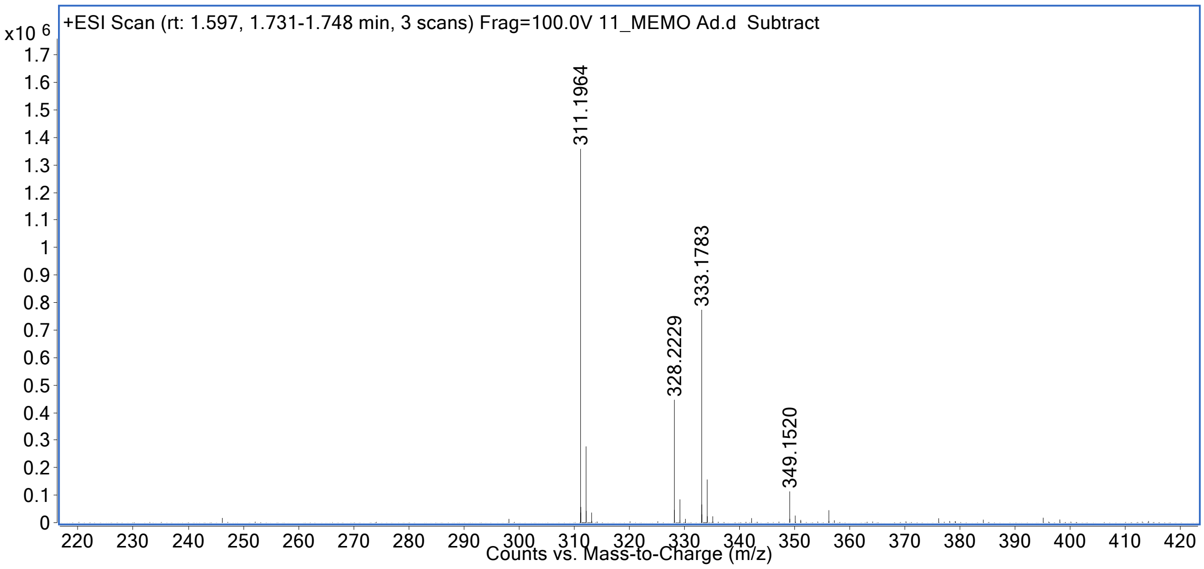

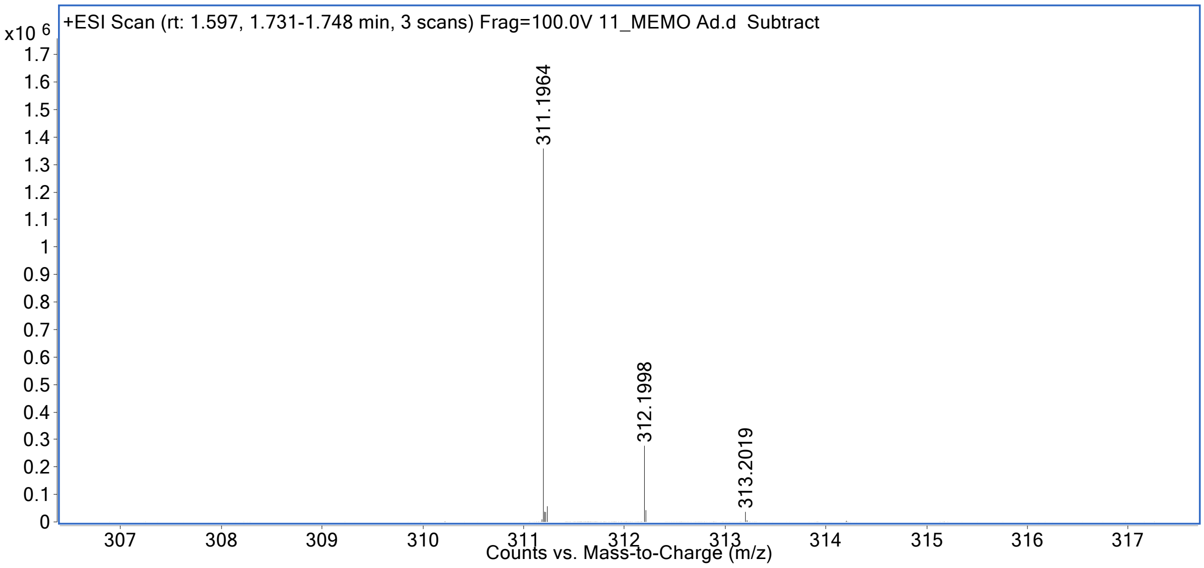

(*E*)-*N*-cyclohexyl-2,5,7-trioxa-8-azadec-8-en-10-amide

Calculated $[M+H]^+$: 259,1652

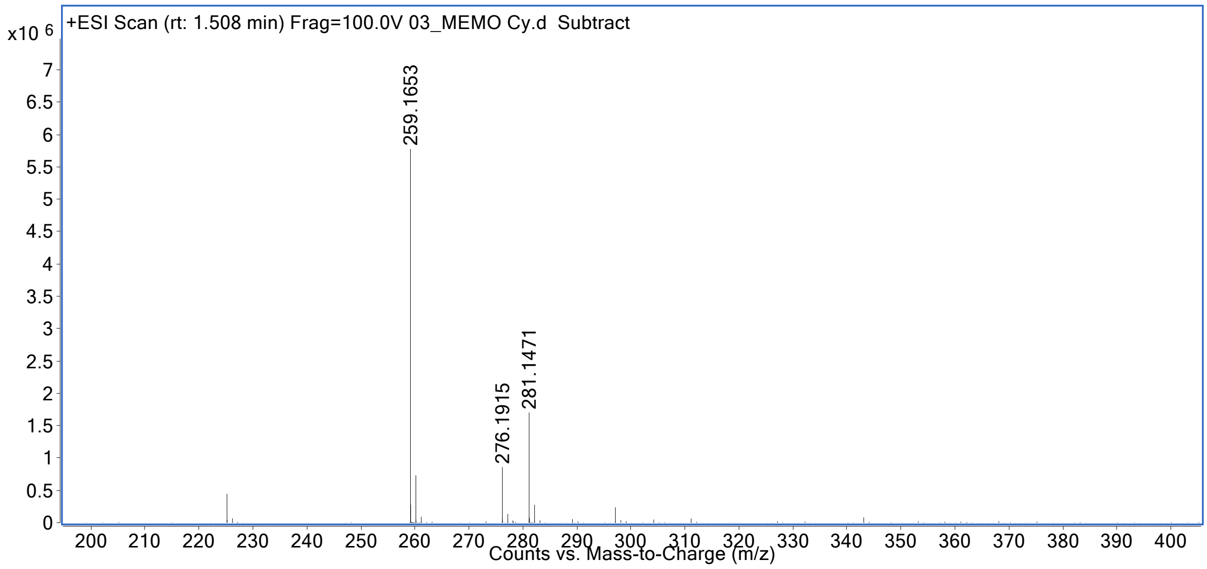

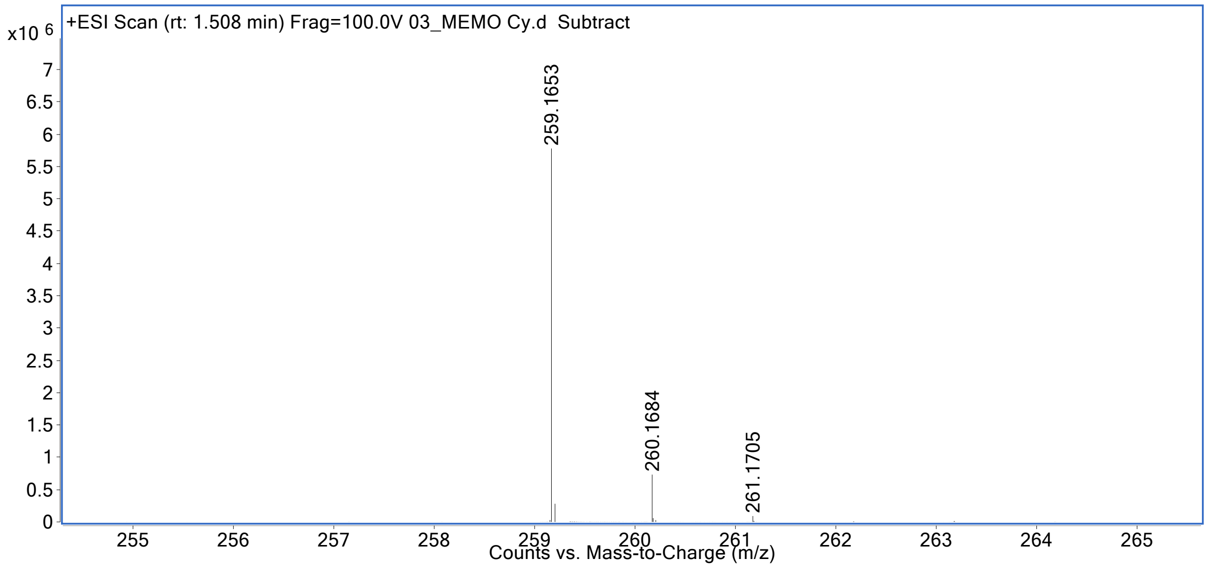

<u>(*E*)-*N*-(*tert*-butyl)-2,5,7-trioxa-8-azadec-8-en-10-amide</u>

Calculated $[M+H]^+$: 233,1496

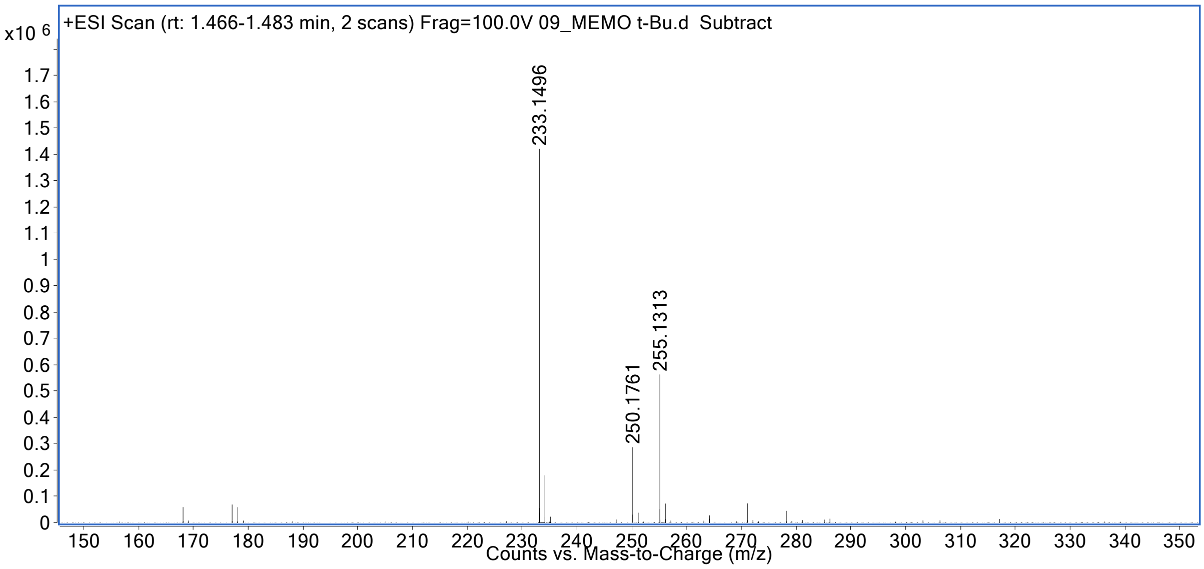

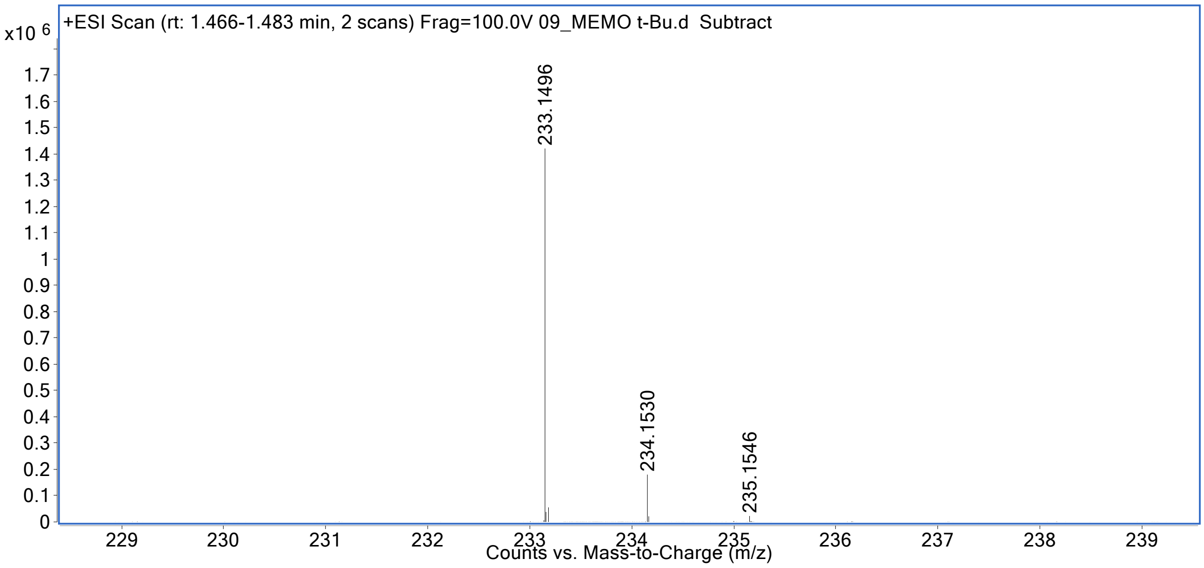

References

[1] S. N. Mantrov, Yu. M. Lapina, E. A. Shukhtina, "New Synthesis of 2-Oxoalkanamide Oximes" *Russ. J. Org. Chem.* **2019**, *55*, 540–545.

[2] K. Thalluri, K. C. Nadimpally, A. Paul, B. Mandal, "Waste reduction in amide synthesis by a continuous method based on recycling of the reaction mixture" *RSC Adv.* **2012**, *2*, 6838.

[3] Md. A. Ali, S. M. A. H. Siddiki, W. Onodera, K. Kon, K. Shimizu, "Amidation of Carboxylic Acids with Amines by $Nb_2 O_5$ as a Reusable Lewis Acid Catalyst" *ChemCatChem* **2015**, *7*, 3555–3561.

[4] M. Nechab, N. Azzi, N. Vanthuyne, M. Bertrand, S. Gastaldi, G. Gil, "Highly Selective Enzymatic Kinetic Resolution of Primary Amines at 80 °C: A Comparative Study of Carboxylic Acids and Their Ethyl Esters as Acyl Donors" *J. Org. Chem.* **2007**, *72*, 6918–6923.

[5] I.-H. Kim, F. R. Heirtzler, C. Morisseau, K. Nishi, H.-J. Tsai, B. D. Hammock, "Optimization of Amide-Based Inhibitors of Soluble Epoxide Hydrolase with Improved Water Solubility" *J. Med. Chem.* **2005**, *48*, 3621–3629.

[6] A. Torihata, C. Kuroda, "Reaction of Amphipathic-Type Thioester and Amine with Hydrophobic Effect in Water" *Synlett* **2011**, *2011*, 2035–2038.

[7] Q. Li, H. Jin, Y. Liu, B. Zhou, "Nickel-Catalyzed Multicomponent Coupling Reaction of Alkyl Halides, Isocyanides and H2O: An Expedient Way to Access Alkyl Amides" *Synthesis* **2020**, *52*, 3466–3472.

[8] B. R. Brooks, R. E. Bruccoleri, B. D. Olafson, D. J. States, S. Swaminathan, M. Karplus, "CHARMM : A program for macromolecular energy, minimization, and dynamics calculations" *J. Comput. Chem.* **1983**, *4*, 187–217.

[9] F. A. Momany, R. Rone, "Validation of the general purpose QUANTA ®3.2/CHARMm® force field" *J. Comput. Chem.* **1992**, *13*, 888–900.

[10] B. Stauch, S. J. Fisher, M. Cianci, "Open and closed states of Candida antarctica lipase B: protonation and the mechanism of interfacial activation" *J. Lipid Res.* **2015**, *56*, 2348–2358.

[11] M. Feig, C. L. Brooks, "Recent advances in the development and application of implicit solvent models in biomolecule simulations" *Curr. Opin. Struct. Biol.* **2004**, *14*, 217–224.

[12] M. Nina, D. Beglov, B. Roux, "Atomic Radii for Continuum Electrostatics Calculations Based on Molecular Dynamics Free Energy Simulations" *J. Phys. Chem. B* **1997**, *101*, 5239–5248.
